# Students’ use of caffeine, alcohol, dietary supplements, and illegal substances for improving academic performance in a New Zealand university

**DOI:** 10.1080/21642850.2021.1990763

**Published:** 2021-10-22

**Authors:** Anantha Narayanan, Malcolm Gill, Chaey Leem, Cassandra Li, Frances Mein Smith, Ben Shepherd, Selene Ting, Karin van Bart, James A. Green, Ari Samaranayaka, Christina Ergler, Alexandra Macmillan

**Affiliations:** a Dunedin School of Medicine, University of Otago, Dunedin, New Zealand; b School of Pharmacy, University of Otago, Dunedin, New Zealand; c School of Allied Health, University of Limerick, Limerick, Ireland; d Physical Activity for Health Research Cluster, Health Research Institute, University of Limerick, Limerick, Ireland; e Department of Preventive and Social Medicine, University of Otago, Dunedin, New Zealand; f Department of Geography, University of Otago, Dunedin, New Zealand

**Keywords:** methylphenidate, New Zealand, cognitive enhancement, performance enhancing substances, universities

## Abstract

This study aimed to describe patterns of use and attitudes towards a broad variety of substances for improving academic performance at a New Zealand university. 685 students (from 1800 invited) completed an online questionnaire (38% response rate). They were asked about their lifetime and current substance use for improving academic performance, as well as their reasons for use, attitudes and perceptions of: caffeine, alcohol, dietary supplements, prescription stimulants, other prescription substances, and illicit substances. 80% (95% CI: 76.3, 82.5) reported ever using any substance to help improve academic performance, mainly to stay awake and improve concentration. Caffeine (70%, 95% CI: 66.3, 73.3) and dietary supplements (32%, 95% CI: 28.3, 35.5) were most commonly used. 4% (95% CI: 2.7, 5.9) reported use of prescription stimulants, mostly methylphenidate, and another 4% (95% CI: 2.7, 5.9) reported using illicit substances for improving academic performance. Users of prescription stimulants were more likely than non-users to believe that they were safe, morally acceptable, and that they should be available legally for enhancing academic performance. We close with discussions on broadening the focus of substances for improving academic performance in public health debates. Further qualitative research from small countries is also needed to move towards a place-based approach for clarifying ethical implications, inform policy in universities, and understand how injustices are created through the use of and ability to purchase different substances.

## 1. Introduction

Substance use to improve academic performance has reached global headlines for the health risks posed by the use of prescription stimulants, and the potential ethical and moral implications (Leonard, McCartan, White, & King, 2004; Simoni-Wastila & Strickler, 2004; Smith & Farah, 2011; Solomon, Adams, Silver, Zimmer, & DeVeaux, 2002). Substances that are used for improving academic performance range from everyday stimulants such as caffeine to the non-medical use of prescription stimulants such as methylphenidate, modafinil, and Adderall (mixed amphetamine salts).

Cognitive enhancement is one way that substance use can improve academic performance. However, substances can also be used to improve performance without altering cognition. Examples include beta-blockers and benzodiazepines, which can be used to manage anxiety. Much prior research has focused narrowly on the use of prescription stimulants (e.g. Leonard et al., 2004; Simoni-Wastila & Strickler, 2004; Smith & Farah, 2011; Solomon et al., 2002), but recent papers have demonstrated that students use a wide range of lifestyle, recreational and illicit drugs to improve their academic performance (e.g. Forlini, Schildmann, Roser, Beranek, & Vollmann, 2015; Maier, Liakoni, Schildmann, Schaub, & Liechti, 2015; Mazanov, Dunn, Connor, & Fielding, 2013; Schelle et al., 2015). This research adds to this growing literature on the broader context of substance use for enhancing academic performance and argues for a place-based approach to better understand the local prevalence and life context of users in order to tailor policies and interventions to the needs of the targeted population.

Substance use for academic purposes may harm health directly, or indirectly. For example, methylphenidate has serious side effects and potential for dependency (Leonard et al., 2004; Simoni-Wastila & Strickler, 2004). These risks are rare in medically-indicated use, but a recent review highlights concerns for non-medical use (Urban & Gao, 2017). Even substances generally considered benign, such as non-vitamin non-mineral supplements, have adverse effects. One study estimated the rates of adverse effects as high as 14% (Newberry, Beerman, Duncan, McGuire, & Hillers, 2001). Moreover, despite high rates of use, there is little research on the long term impacts of common energy drink ingredients (Breda et al., 2014). Forlini and colleagues (2015) even suggest that non-prescription stimulants may be a more immediate and important area that needs interventions from a public health perspective, especially as students who use substances to enhance academic performance are also more likely to use illicit drugs and engage in risky drink-driving behaviors (McCabe, Knight, Teter, & Wechsler, 2005).

There are also legal and ethical implications about improving academic performance. The use of cognitive enhancers and associated ideas about ‘good’ or ‘bad’ behavior, is therefore important to consider and speaks to wider debates on the medicalization and pharmaceuticalization of social life (Petersen, Nørgaard, & Traulsen, 2015; Williams, Martin, & Gabe, 2011). For example, DeSantis and Hane’s (2010) participants perceived the misappropriation of Adderall for study purposes as a safe and morally acceptable form of self-medicalization, whereas Partridge, Bell, Lucke, and Hall (2013) report concerns of side-effects for long-term users and variations in effectiveness due to people responding differently to drugs.

Prevalence of self-reported substance use for improving academic performance in tertiary students varies between and across countries and gender. For prescription stimulants, estimates in the United States range from 6.9% (McCabe et al., 2005) to 16% (White, Becker-Blease, & Grace-Bishop, 2006). Reports for Switzerland are also high (12%, Maier et al., 2015), in contrast with Australia (2.1-4.0%, Mazanov et al., 2013; Paliza, 2011; Riddell, Jensen, & Carter, 2018), Germany (2%, Forlini et al., 2015; Mache, Eickenhorst, Vitzthum, Klapp, & Groneberg, 2012), and the Netherlands (1.7%, Schelle et al., 2015). In studies where gender contrasts are made, men usually have higher prevalence than women (Hall, Irwin, Bowman, Frankenberger, & Jewett, 2005; McCabe et al., 2005).

Higher prevalence estimates in the US may have two drivers: many studies there consider non-medical use of prescription stimulants (e.g. Hall et al., 2005; McCabe et al., 2005; White et al., 2006), which is broader than use *only* for cognitive enhancement. Further, availability and access to methylphenidate is higher in the US, with diversion and abuse of prescription stimulants more commonly reported in countries that have a high consumption (International Narcotics Control Board, 2013). In 1992, 86% of all methylphenidate manufactured was consumed in the US, dropping to 69% by 2011, as use increased in countries such as Belgium, Spain, Australia and New Zealand (International Narcotics Control Board, 2013). The only New Zealand study looking at substance use for improving academic performance was based on a convenience sample of students from professional courses, and findings suggest a prevalence similar to the lower end of the US range at 6.6% (Ram, Hussainy, Henning, Jensen, & Russell, 2015). However to get a better picture of the use and perceptions of cognitive enhancement substances more studies from different university contexts and study programs within New Zealand are needed.

While there are clear differences in the prevalence between and within countries, the evidence regarding the efficacy of various substances for improving academic performance is inconclusive. A review of 28 studies on the non-medical use of prescription stimulants tentatively supports their positive effects on consolidation of fact-based learning, but results were mixed concerning working memory, cognitive control, and executive function (Smith & Farah, 2011). In particular qualitative studies suggest diverse experiences with substances and varying effects on academic performance, but point to the need to understand the life context of users (Forlini & Racine, 2012; Hildt, Lieb, & Franke, 2014; Partridge et al., 2013; Petersen et al., 2015; Sharif, Guirguis, Fergus, & Schifano, 2021). Overall, studies reviewed were difficult to compare due to their varied study designs, and publication bias could exaggerate the enhancement potential of stimulants. It is also likely that dietary supplements (e.g. fish oil, *Ginkgo biloba*) do not achieve their claims of improving academic performance. For example, a double-blinded randomized controlled trial showed that *Ginkgo biloba* did not appear to improve memory or other related cognitive function (Solomon et al., 2002).

Nonetheless, commonly reported reasons for using cognitive enhancing drugs include staying awake, improving concentration and alertness, keeping up, getting ahead, coping with stressful tasks, and increasing efficiency (DeSantis & Hane, 2010; Hildt et al., 2014; Mache et al., 2012; Mazanov et al., 2013; Parks et al., 2017; Partridge et al., 2013; Petersen et al., 2015; Riddell et al., 2018; Teter, McCabe, Cranford, Boyd, & Guthrie, 2005). Some students do not believe prescription stimulants result in improvements in academic performance, and also identify possible adverse effects including addiction/dependency, mental health issues, and sleep disturbance (Forlini & Racine, 2009, 2012; Franke, Lieb, & Hildt, 2012; Judson & Langdon, 2009; Partridge et al., 2013). Students who use prescription stimulants to aid concentration report that this behavior is more ethical compared to non-users (Judson & Langdon, 2009; Petersen et al., 2015). Students who do not use such substances to enhance their academic performance are therefore more likely see this behavior as unethical or ‘cheating’.

We therefore further recent research (Forlini et al., 2015; Maier et al., 2015; Mazanov et al., 2013; Schelle et al., 2015), by demonstrating that substance use for academic purposes involves a much broader range of substances than merely prescription stimulants. We also examine the extent to which students using one type of substance also use other substances. We also explore the underlying reasons, attitudes and moral and risk perceptions surrounding the use of diverse freely available and over-the-counter substances for improving academic performance. Finally, we will add to and broaden the context of the only existing estimate for New Zealand by adding more accurate estimates based on a random probability sample to the existing discussion. As the study is located in a different New Zealand university environment with a residential campus and the majority of students living within walking distance to university, often referred to as ‘Studentville’, we can add to a more place-based understanding of prevalence within a country. Moreover, we can also add to debates on differences in use between competitive and less competitive programs (e.g. law, health sciences and humanities), to consider whether students in high-pressure, high-stress programs might be more likely to actively manage their workload with substance use for academic purposes.

## 2. Material and methods

We undertook a random, anonymous, cross-sectional, online prevalence survey of undergraduate and postgraduate tertiary students in a single, publicly funded New Zealand University (student population approximately 20,600).

### 2.1. Participants

Our sampling frame was students over the age of 18 years, currently enrolled at the University of Otago and studying at the Dunedin campus in New Zealand. Using Ram et al.’s (2015) New Zealand prevalence estimate of 6%, to get a precision of 2% (i.e. half width of 95% CI) we required approximately 600 participants in the sample. Using a worst case response rate of 33% we invited a sample of 1800, selected from enrollment records across all degrees using electronic uniform random number generation. In September 2014, an initial email was sent to these students; it provided information about the online survey and invited students to participate via a link to the online survey. The survey was open for three weeks. To maximize the response rate two further reminder emails were sent six days apart. Upon completion of the survey, participants were offered the option to enter an anonymous draw to win one of four supermarket vouchers (details collected on a separate, unlinked website). This research protocol was approved by the University of Otago Human Ethics Committee (F14/011), with all participants completing an online written consent.

Of the 1800 students invited to participate, 719 consented to participate, giving a response rate of 40%. Thirty-four of those responses (4.7%) were then excluded because of incomplete data, leaving 685 responses, so we report results for 38% of those invited to participate. As Table 1 illustrates, the sample was generally representative of the student population, but with fewer men relative to women, and older students (27+ years) less well represented.

**Table 1.** Characteristics of the sample, and comparison with University of Otago student population.

		Participants			Population	
		n	%	95% CI	n	%
Gender^1^						
	Male	227	33	(30, 37)	8,720	42.3
	Female	458	67	(63, 70)	11,879	57.7
Age^2^						
	18–20	338	49	(46, 53)	9,003	43.7
	21–23	240	35	(31, 39)	5,868	28.5
	24–26	51	7	(5, 9)	1,716	8.3
	27+	56	8	(6, 10)	3,911	19.0
Ethnicity^3^						
	NZ European	535	78	(75, 81)	15,126	73.4
	Māori	47	7	(5, 9)	1,759	8.5
	Pacific	18	3	(1, 4)	804	3.9
	Asian	105	15	(13, 18)	3,877	18.8
	Other	36	5	(4, 7)	14,88	7.2
Course^3^						
	Bachelor	536	78	(75, 81)	15,105	72.1
	Undergraduate diploma	43	6	(4, 8)	47	2.1
	Postgraduate diploma/certificate	23	3	(2, 5)	1,366	6.5
	Masters	23	3	(2, 5)	1,196	5.7
	Honors	30	4	(3, 6)	431	2.1
	PhD	30	4	(3, 6)	1,328	6.3
Academic divisions^4^						
	Humanities	207	30	(27, 34)		26.8
	Business	83	12	(10, 15)		15.9
	Science	225	33	(29, 36)		24.0
	Health Sciences	228	33	(30, 37)		31.9
	Other	42	6	(4, 8)		
Total		685			20,601	

Notes: ^1^2 students identified as gender diverse.

^2^ 103 students were under the age of 18 in the student population, however would have been excluded from answering the survey questionnaire.

^3^ Multiple responses allowed.

^4^ Multiple responses were allowed for participants. However, population percentages are Equivalent Full-time Students per division.

### 2.2. Materials

We used an online survey divided into three sections: demographics, substance use, and attitudes. Demographic information included age, gender, self-identified ethnicity, level of study (Bachelors, Honors, Masters, etc.), current year of study, course of study, international/domestic student status, and accommodation (shared house, own home, residential college, etc.). The full text of the survey is included as Appendix A.

The survey was co-designed with the student authors on the paper to ensure the language and diverse substances queried spoke to the language and potential use of the current student cohort. Further, to reduce ambiguity about the purpose of different substances, following Forlini et al. (2015) we provided a clear and focused description of situations in which substances can be purposefully used for academic enhancement at the beginning of the questionnaire. The substances of interest were divided into six groups and presented to participants as follows: *caffeine* (e.g. drinks containing caffeine, caffeine tablets), *alcohol*, *dietary supplements* (e.g. vitamins, herbal supplements and herbal teas), *prescription stimulants* (e.g. methylphenidate/Ritalin/Concerta, Adderall/mixed salt amphetamines), *other prescription medicines* (e.g. diazepam/Valium, betablockers, codeine, Oxynorm, Oxycontin, morphine), and *illicit drugs* (e.g. cannabis, cocaine, methamphetamine, LSD, ecstasy, GHB, magic mushrooms, heroin).

For each group of substances, participants were asked whether they had ever used a substance specifically for the purpose of improving academic performance; for example, this could be to improve concentration, alertness, relaxation (see also Forlini et al., 2015). Participants that had used a substance within the last six months were considered current users, and were asked further questions about their substance use in the last six months. These included information about: the specific substances used; frequency of substance use; timing and reasons for use. Participants were also asked about their recreational drug use.

Additional information was obtained regarding the use of prescription stimulants and other prescription substances. These questions were: frequency of substance use in the past, but excluding in the last six months; and whether these substances were used for a diagnosed medical condition (e.g. methylphenidate use for attention deficit hyperactivity disorder).

All participants were asked about their attitude towards the use of substances to improve academic performance. For each group of substances, participants were asked to rate how strongly they agreed or disagreed on a 5 point Likert scale with statements regarding the safety and morality of its use to improve academic performance. There were additional questions about attitudes towards prescription stimulant use. Participants were also asked whether they believed prescription stimulants were: addictive; effective at improving academic performance; if their use is common practice among students at the University of Otago; and whether they should be made legally available for the purpose of improving academic performance.

### 2.3. Data analysis

Respondents who identified as having been prescribed stimulants to treat a medical diagnosis were excluded from analysis of prescription stimulants. The number of respondents that reported using prescription stimulants without a medical diagnosis, other prescription medicines (with or without a medical diagnosis) or illicit drugs was relatively low. For this reason, they were analysed together as ‘controlled substances’. Courses of study were grouped into their respective academic divisions (humanities, business, science, health sciences), with three large, high-workload, more-competitive programs analysed separately. These programs were Bachelor of Law, Health Sciences First Year (HSFY, a common competitive year for students attempting to enter health professional courses including medicine), and the Bachelor of Medicine and Bachelor of Surgery (medical degree) itself.

We estimated the prevalence of use of each group of substances across demographic categories using list-wise deletion of participants with missing data. Logistic regression was used to estimate odds ratios for relative prevalence across different demographic groups. Since expected prevalence of substance use was low, these odds ratios were assumed to be approximations for relative risks of substance use in order to identify subgroups with higher prevalence of use. We used Stata 13.1 and SPSS 23 software for the analyses.

## 3. Results

### 3.1. Prevalence

Eighty percent of all respondents reported ever using at least one substance to help them improve their academic performance, though by far the most common substance used was caffeine (70% of participants, see Table 2). About a third of students had used dietary supplements. Ten percent of students reported ever using any controlled substance (prescription stimulants, other prescription medicines, illicit drugs), with 6% reported they had used one of these substances in the last six months. Four percent of participants reported having ever used prescription stimulants with 2% having used it in the past six months. Methylphenidate was the most commonly reported prescription stimulant. Table B.1 shows concurrent usage in the last 6 months: most reported being current users of one substance (caffeine), but where students reported two, this was most likely one other substance along with caffeine (overlap 66-94%). Prescription stimulant users were likely to use other prescription substances, but there was no overlap between prescription stimulants and illicit substances.

**Table 2.** Prevalence of use by substance for improving academic performance.

Substance	Ever used			Used in the last 6 months		
	*n*	%	95% CI	*n*	%	95% CI
Caffeine	479	70	(66.3, 73.3)	416	61	(57, 64.4)
Alcohol	42	6	(4.5, 8.2)	25	4	(2.4, 5.3)
Dietary supplements	218	32	(28.3, 35.5)	161	24	(20.4, 26.9)
Prescription stimulants^1^	28	4	(2.7, 5.9)	11	2	(0.8, 2.9)
Other prescription substances	19	3	(2.7, 4.3)	15	2	(1.2, 3.6)
Illicit substances	28	4	(2.7, 5.9)	19	3	(1.7, 4.3)
Controlled substances^2^	67	10	(7.7, 12.3)	42	6	(4.5, 8.2)
Any substance	545	80	(76.3, 82.5)	481	70	(66.6, 73.6)

Notes: ^1^Excludes people with a diagnosis of ADHD.

^2^ Prescription stimulant, other prescription substance and illicit substance combined.

A number of demographic variables were associated with lifetime prevalence (Table 3). Women had lower prevalence of alcohol use, prescription stimulants and illicit drugs. For almost all substance classes, we found increasing use with age and year of study, tailing off for the most senior students, though we have relatively few participants in the higher age and year categories. The exception to this pattern was in the use of dietary supplements where the opposite pattern was evident.

**Table 3.** Univariate odds ratios showing relationship between demographic factors and lifetime use of substances.

	CaffeineOR (95% CI)	Alcohol OR (95% CI)	Dietary Supplements OR (95% CI)	All controlled substances OR (95% CI)
Gender				
Male				
Female	0.99(0.70-1.40)	**0.52(0.28-0.98)**	1.29(0.91-1.83)	**0.32**(**0.19-0.54)**
Age (yrs)				
18-20	(ref.)			
21-23	**1.54**(**1.06-2.23)**	1.73(0.87-3.44)	1.18(0.83-1.69)	1.52(0.84-2.77)
24-26	**2.34**(**1.10-4.98)**	1.71(0.55-5.34)	1.14(0.61-2.13)	**2.54**(**1.07-6.05)**
27+	0.62(0.35-1.10)	1.14(0.32-4.04)	0.91(0.49-1.70)	**2.62**(**1.14-6.01)**
Year of study				
First	(ref.)			
Second	**1.61**(**1.01-2.58)**	1.66(0.59-4.67)	1.05(0.64-1.70)	1.56(0.59-4.14)
Third	**2.10**(**1.31-3.36)**	1.02(0.33-3.10)	1.21(0.76-1.93)	2.10(0.84-5.24)
Fourth	**2.06**(**1.18-3.60)**	2.17(0.73-6.47)	1.18(0.68-2.03)	2.34(0.87-6.44)
Fifth	**2.68**(**1.31-5.48)**	**3.38**(**1.09-10.55)**	0.74(0.37-1.49)	**4.38**(**1.58-12.14)**
6th	2.86(0.78-10.57)	3.72(0.68-20.31)	0.57(0.15-2.12)	3.17(0.60-16.83)
7+	1.63(0.75-3.55)	1.42(0.27-7.35)	1.29(0.60-2.77)	**5.88**(**1.97-17.52)**
Subject Area^1^ (multiple subject areas possible)				
Humanities	1.19(0.83-1.71)	1.80(0.96-3.40)	1.07(0.75-1.52)	**2.06**(**1.22-3.48)**
Business	1.07(0.64-1.77)	1.17(0.02-1.23)	0.80(0.48-1.33)	1.21(0.57-2.55)
Sciences	0.91(0.65-1.27)	0.90(0.45-1.73)	1.23(0.88-1.71)	0.74(0.42-1.29)
Health Sci.	0.97(0.68-1.38)	0.71(0.34-1.48)	0.86(0.61-1.23)	0.57(0.30-1.06)
Competitive courses^1^				
Medicine	1.13(0.67-1.90)	0.80(0.28-2.30)	0.65(0.38-1.11)	1.10(0.51-2.42)
Law	1.11(0.64-1.91)	1.81(0.77-4.24)	0.96(0.56-1.63)	1.48(0.70-3.13)
HSFY	0.62(0.33-1.16)	0.70(0.16-2.98)	0.86(0.44-1.68)	no users

Note: Bolded values indicate CI does not include 1.0. All analyses include only a single predictor (e.g. gender, age, course of study).

^1^ For these analyses, each subject course group was compared to all other students in separate analyses, due to potentially overlapping categories.

No students in our sample studying in the competitive entry year into health professional courses (Health Science First Year) reported using any controlled substance for academic purposes. Non-stimulant prescription medicines were less used by 18–20 year olds. Humanities students were more likely to have used illicit drugs for study purposes. Table B.2 presents parallel analyses for use in the last 6 months. Patterns for last 6 months use were largely similar to lifetime use.

### 3.2. Timing of use

As outlined in Table 4, around a third of participants used caffeine at least once a day, and 15% used dietary supplements daily. All other substances were used very infrequently. The most common times for using all substances was before assignment deadlines and exams (full details in Table B.3).

**Table 4.** Frequency of use for improving academic performance by substance.

Frequency of use (for any purpose)	Caffeine	Alcohol	Dietary supplements	Prescription stimulants	Other prescription substances	Other illicit substances
	% (95% CI)	% (95% CI)	% (95% CI)	% (95% CI)	% (95% CI)	% (95% CI)
At least once a day	32 (29, 36)	0 (0, 0)	15 (12, 18)	0 (0, 0)	1 (0, 1)	1 (0, 1)
Once a week	16 (13, 18)	1 (0, 1)	5 (4, 7)	0 (0, 0)	0 (0, 1)	1 (0, 2)
1–2 times a month	7 (5, 9)	1 (0, 2)	2 (0, 0)	0 (0, 1)	0 (0, 1)	1 (0, 1)
Less than once a month	6 (4, 7)	1 (0, 2)	1 (0, 2)	1 (0, 2)	1 (0, 1)	0 (0, 1)
Used, but not in last 6 months	10 (7, 12)	3 (2, 4)	9 (6, 11)	3 (1, 4)	1 (0, 1)	1 (0, 2)
Never used	30 (27, 34)	94 (92, 96)	68 (65, 72)	96 (95, 98)	97 (96, 98)	96 (95, 98)

### 3.3. Reasons for use

Of all participants who used any substance, the most common reasons were to stay awake or improve concentration (full details in Table B.4). Almost all respondents who took prescription stimulants did so to improve concentration. However, a substantial number of students also used alcohol, illicit drugs and non-stimulant prescription drugs for relaxation and to manage stress.

### 3.4. Perceptions of effectiveness, safety and morality

Most respondents considered caffeine and dietary supplements to be safe and morally acceptable for improving academic performance. Fewer people considered prescription stimulants, other prescription substances and illicit drugs to be safe and moral for this purpose (Table 5). Users of prescription stimulants were more likely than non-users to consider the use of prescription stimulants for improving academic performance to be safe (OR 67.1, *95% CI* 8.6, 524.2) and morally acceptable (OR 9.1, *95% CI* 2.6, 31.9). In response to specific questions about methylphenidate/Ritalin, more than half of respondents were unsure whether it was addictive (59%), effective at improving academic performance (57%), or common practice at the University of Otago (57%). Most disagreed that Ritalin should be legally available for the purpose of improving academic performance (58%). However, current users of prescription stimulants were more likely to agree that Ritalin should be legal for the purpose of improving academic performance (OR 12.5, *95% CI* 4.1-37.8).

**Table 5.** Perceptions of safety and morality of substances for improving academic performance.

	Strongly agree	Agree	Neither agree nor disagree	Disagree	Strongly disagree
	% (95% CI)	% (95% CI)	% (95% CI)	% (95% CI)	% (95% CI)
**Safety**					
Use of caffeine is safe	23 (19, 26)	57 (53, 60)	16 (13, 19)	4 (3, 6)	1 (0, 2)
Use of dietary supplements is safe	24 (21, 28)	43 (39, 46)	28 (25, 32)	4 (2, 5)	2 (1, 3)
Use of prescription stimulants is safe	2 (1, 3)	10 (8, 12)	33 (29, 36)	34 (30, 37)	22 (19, 25)
Use of other prescription substances is safe	0 (0, 1)	4 (2, 5)	30 (27, 34)	35 (31, 38)	31 (28, 35)
Use of illicit substances is safe	0 (0, 1)	2 (1, 4)	12 (10, 15)	23 (20, 26)	62 (59, 66)
**Morality**					
Use of caffeine is moral	38 (35, 42)	42 (38, 46)	16 (13, 19)	2 (1, 4)	2 (1, 3)
Use of dietary supplements is moral	35 (31, 38)	38 (35, 42)	22 (19, 25)	3 (2, 5)	2 (1, 3)
Use of prescription stimulants is moral	5 (3, 7)	8 (6, 11)	24 (21, 27)	29 (26, 33)	34 (31, 38)
Use of other prescription substances is moral	4 (3, 6)	5 (4, 7)	22 (19, 25)	29 (25, 32)	40 (37, 44)
Use of illicit substances is moral	4 (3, 6)	4 (3, 6)	16 (14, 19)	20 (17, 23)	56 (52, 60)
**Ritalin (methylphenidate)**					
Ritalin is addictive	11 (8, 13)	26 (23, 30)	59 (56, 63)	3 (2, 5)	1 (0, 2)
Ritalin is effective at improving academic performance	7 (6, 10)	25 (21, 28)	57 (54, 61)	7 (5, 9)	4 (2, 5)
Ritalin use is common among [institution] students	4 (2, 5)	14 (11, 17)	57 (54, 61)	19 (16, 22)	6 (5, 9)
Ritalin should be legally available	2 (1, 3)	5 (4, 7)	34 (31, 38)	25 (22, 29)	33 (30, 37)

## 4. Discussion

Most students had used one or more substances – mostly caffeine or dietary supplements – for improving academic performance. Similar findings have been reported for German university students in the Ruhr area who used mainly caffeine and energy drinks to improve their concentration and stay awake (Forlini et al., 2015). Current use of prescription stimulants was low in line with most international data (Mache et al., 2012; Mazanov et al., 2013; Paliza, 2011; Schelle et al., 2015), but lower than the only previous estimate for New Zealand (Ram et al., 2015) as well as estimates for the United States (Hall et al., 2005; McCabe et al., 2005), and Switzerland (Maier et al., 2015). Some previously reported US studies and the prior New Zealand study may report greater prevalence estimates because they asked about any ‘non-medical’ use of prescription stimulants, which will include recreational use. As noted earlier, wider availability and access to methylphenidate is also a likely driver for higher rates of use in the US (Kieffer, Cronin, & Gawet, 2006; Vuckovic, 1999). These findings indicate the need to be very clear about the context and aim of using a wide range of cognitive enhancement stimulants (see also Forlini et al., 2015). Moreover, these studies also do not distinguish between competitive and less competitive programs, as was the case in the prior New Zealand study, which drew their sample only from competitive programs. High prevalence of stimulants has been observed in medical students for example (De Bruyn, Wouters, Ponnet, & Van Hal, 2019), and at more competitive institutions (McCabe et al., 2005). Our data shows that for understanding the differences in prevalence a broader sample is required as well as the need to conduct more studies that address the local context of studying. For instance, how the living arrangements of students (e.g. residential college, shared housing) influence their use of some substances. Given the differences within and between countries (Sharif et al., 2021) our study points towards the need to take a place-based approach for understanding the differences in prevalence. We envision that for our ‘place’, that such an approach includes the local student culture and dominant student identities, but also other factors such as the studentification of the campus area, study background of participating students, life aspirations and societal attitudes towards cognitive enhancers and the accessibility of enhancers (Petersen, Lyngsø-Dahl Ølgaard, & Nørgaard, 2019; Petersen, Petersen, Poulsen, & Nørgaard, 2021; Ram et al., 2017; Ram et al., 2021).

We investigated substance use ‘with the goal of improving academic performance’ – which is not as restrictive as ‘for cognitive enhancement’ (e.g. Mache et al., 2012) but also not as broad as ‘for non-medical use’ (e.g. McCabe et al., 2005). This meant that we were able to ask about a spectrum of substances that would not generally be thought of as ‘cognitive enhancers’, but at the same time excluding recreational use (for example, alcohol, prescription medicines not classed as stimulants, and illicit drugs such as cannabis). Despite this inclusive definition, the majority of students still reported a wide variety of substance use with the approach to enhance cognition, but students also highlighted the need to use stimulants for coping with university life (see Hildt et al., 2014; Petersen et al., 2015; Riddell et al., 2018). There is little research regarding the use of these substances in the context of improving academic performance (Forlini et al., 2015; Kieffer et al., 2006; Moser, Dellen, & Lundt, 1979). Only a small number of students identified using prescription medicines (excluding stimulants), illicit drugs, and alcohol for the purpose of improving academic performance. In contrast, we found caffeine to be used by the greatest proportion of participants – most commonly coffee or other caffeinated beverages rather than tablets or over the counter preparations – along with dietary supplements. These findings are in line with previous reports (Ambrose & Samuels, 2004; Dundas & Keller, 2003; Johnson & Blanchard, 2006; Lee et al., 2009; Mache et al., 2012; Mazanov et al., 2013; Newberry et al., 2001; Paliza, 2011; Perkin, Wilson, Schuster, Rodriguez, & Allen-Chabot, 2002; Riddell et al., 2018; Simoni-Wastila & Strickler, 2004; Stasio, Curry, Sutton-Skinner, & Glassman, 2010). In terms of concurrent use, we found that those who used prescription stimulants were not likely to also use illicit substances for improving academic performance. This contrasts with the findings of Schelle et al. (2015). We speculate that it may be a difference in source, possibly with prescription stimulants acquired through diversion of friends’ prescriptions in New Zealand, meaning less overlap between prescription and illicit drug use. In a recent survey of New Zealand psychiatrists, 20% reported receiving requests from university students for cognitive enhancers (Ram et al., 2021). Improving concentration was one of the most commonly stated reasons for using all substances, except alcohol. Dietary supplements were commonly used to improve memory, despite limited to no evidence for their efficacy. Compared with other substances, prescription stimulants were more commonly used for ‘managing the pressure to succeed’, which has also been reported previously (Coveney, 2011; Forlini & Racine, 2012; Hildt et al., 2014). This may indicate an area for targeted interventions aimed at decreasing perceived pressure by students, which we will discuss in more detail below. In contrast, students did not identify peer pressure as an influence; no users of any controlled substances and alcohol selected ‘because others use it’ as a reason for their use.

There was a substantial difference between users and non-users with regards to their perceptions of the safety and morality of use of the various substances for improvement of academic performance. Over a third of our respondents believed methylphenidate to be addictive. Users of each substance were much more likely to consider the use of this substance to be both safe and moral, compared to non-users of the substance. Other studies have shown, although there is some ambiguity reported, that most non-medical users for prescription stimulants were aware of stimulant side effects, as well as some risk of dependency, and tended to be wary of its long-term effects (Forlini et al., 2015; Franke et al., 2012; Partridge et al., 2013). However, non-medical users also appeared to be less concerned about these health risks (Judson & Langdon, 2009). In terms of morality, previous studies have also shown non-medical users of prescription stimulants to be more likely than non-users to consider this use to be ethical (Butcher, 2003; Judson & Langdon, 2009). While the safety and perception of use of prescription stimulants has been widely discussed (Forlini & Racine, 2009; Hildt et al., 2014; Schelle et al., 2015), less is known about the safety and perception of use for the other substances in our study. For example, there is increasing concern about the negative health effects of energy drinks generally (Breda et al., 2014) and for students (Forlini et al., 2015), but interventions hardly target these stimulants, although students turn to energy and caffeinated drinks more frequently (see also Forlini et al., 2015; Riddell et al., 2018). Nonetheless, thirty-two percent of our respondents believed methylphenidate to be effective for improving academic performance, despite the evidence for its efficacy being equivocal (Smith & Farah, 2011). There seems to be a continuum between substances such as caffeine that are clearly considered ethical to use, through to substances that are not considered acceptable. Future research could attempt to more fully illustrate how this is constructed – for example, is it about equality of access? Do students start out trying dietary supplements and move on to controlled substances (a ‘gateway’ effect)? In competitive athletes, users of dietary supplements are more likely to use performance enhancing substances (Backhouse, Whitaker, & Petróczi, 2013). We have future qualitative research planned with student users of prescription stimulants to explore such issues.

Our findings point to a number of areas for intervention, but also further research. First, students in this study and the only other New Zealand study reported to engage in the substance use for academic purposes to deal with study pressures. We speculate that this use may increase due to access barriers decreasing (e.g. via the internet, Petersen et al., 2021), but also a shift in moral values and wider acceptance (Garasic & Lavazza, 2016; Petersen et al., 2019). However, New Zealand universities are not prepared for this scenario. Anecdotal evidence suggests that in New Zealand teaching staff are mostly not aware of substance use for improving academic performance or the risks associated with different substances (e.g. the relationship between anxiety and caffeine) and in particular unintended use of prescription drugs. It is equally absent in university conduct and academic integrity policies. Therefore, substance use for improving academic performance, in particular controlled and illicit substances, is an issue for which it is necessary to raise awareness first among teaching staff. Through their regular interactions with the student body, they are in a good position to alert students to risks and limited evidence of efficacy, but also to address and potentially counteract the actual and perceived pressures students are under. They could, for example, change assessment structures (e.g. fewer but higher weighted internal assessments, moving towards fully internally-assessed courses) and signal their knowledge, understanding, and awareness of the factors impinging on student wellbeing. Some qualitative studies already point towards the perceived increase in using cognitive enhancers in relation to neoliberal policies and the individualization of responsibilities for a successful career (Forlini & Racine, 2009, 2012). Therefore, two approaches should be considered. First, appropriate university policies should be developed that speak to a particular university culture and take into account wider societal debates in a particular country. Forlini and colleagues (2012) discuss the need to develop policies for different stakeholders, but our research suggest that one universal policy is unable to speak to differences within university programs, and given the differences reported within and between countries (Sharif et al., 2021), we suggest that there is a need for place-based policies that are culturally and socially appropropriate. Therefore, more qualitative and quantitative research is needed that addresses the particular ethical and moral understandings of different populations groups (e.g. student users and non-users, teaching staff, medical professionals). Second, information should be made available to students and teaching staff in various forms (e.g. posters, information evenings, social media posts) that speak to their interests, availability and time pressures; and discusses and allows room for questions to address the uncertainty of effects, potential risks and safety debates as well as the justice in relation to use. Moreover, any high-risk groups identified in this or future research should be targeted with appropriate pastoral care and education on alternative coping strategies. However, to tailor these information campaigns more place-based quantitative and qualitative studies are necessary that reveal the knowledge, moral understandings and acceptability of different substances in diverse programs, university cultures and societies.

These findings then can also be used to alter our perceptions of societal norms and increased societal disparity are potential consequences of more widespread use of substances to improve academic performance in the future, regardless of the legality of the practice.

### 4.1. Limitations

Our survey was powered to identify lifetime prevalence across the population, making our estimates of prevalence most useful for highlighting the existence of controlled substance use for improving academic performance in New Zealand Universities. The corollary of this is that there is lower power for exploring demographic sub-groups, with some small cell counts. Therefore, associations with age and year of study, as well as between different courses of study will require more detailed qualitative and quantitative studies with larger sample sizes to confirm these relationships. Future research could also investigate further actual and perceived efficacy of substances used for improving academic performance, as well as investigate how students access these substances.

Selection bias is likely to have affected our prevalence estimates, especially because of the socially sensitive behaviors targeted (Connor, Cousins, Samaranayaka, & Kypri, 2014). However, the demographic characteristics of our sample were similar to the population from which they were drawn. A notable exception was the gender imbalance in our sample (female predominance), though this is common to many studies in this area (Judson & Langdon, 2009; Ram et al., 2015) and more generally. Given that the prevalence of controlled substance use was greater among males, our undersampling of males may have led us to underestimate this prevalence.

### 4.2. Conclusion

We found a wide variety of substances were used for improving academic performance, with caffeine and dietary supplements the most common. Other substances, including alcohol, and controlled substances were used by a very small proportion of students. For prescription stimulants — the focus of much prior research — the lifetime prevalence of use was found to be 4%. This quantification is an important step in developing our understanding of this issue in New Zealand. This study has been effective in generating hypotheses for further research, in terms of possible demographic associations and risk factors for use and the need for a more place-based approached to studying a diverse variety of cognitive enhancements stimulants for improving academic performance and coping with university life.

## Supplementary Material

Supplemental Material

Supplemental Material

## References

[CIT0001] Ambrose, E. T., & Samuels, S. (2004). Perception and use of herbals among students and their practitioners in a university setting. *Journal of the American Academy of Nurse Practitioners*, *16*(4), 166–173. 15137475 10.1111/j.1745-7599.2004.tb00438.x

[CIT0002] Backhouse, S. H., Whitaker, L., & Petróczi, A. (2013). Gateway to doping? Supplement use in the context of preferred competitive situations, doping attitude, beliefs, and norms. *Scandinavian Journal of Medicine and Science in Sports*, *23*(2), 244–252. doi:10.1111/j.1600-0838.2011.01374.x 22092778

[CIT0003] Breda, J. J., Whiting, S. H., Encarnação, R., Norberg, S., Jones, R., Reinap, M., & Jewell, J. (2014). Energy drink consumption in Europe: A Review of the risks, adverse Health effects, and Policy options to respond. *Frontiers in Public Health*, *2*(134), doi:10.3389/fpubh.2014.00134 PMC4197301 25360435

[CIT0004] Butcher, J. (2003). Cognitive enhancement raises ethical concerns. *The Lancet*, *362*(9378), 132–133. doi:10.1016/S0140-6736(03)13897-4 12867135

[CIT0005] Connor, J., Cousins, K., Samaranayaka, A., & Kypri, K. (2014). Situational and contextual factors that increase the risk of harm when students drink: Case–control and case-crossover investigation. *Drug and Alcohol Review*, *33*(4), 401–411. doi:10.1111/dar.12172 24980886

[CIT0006] Coveney, C. M. (2011). Cognitive enhancement? Exploring modafinil use in social context. In M. Pickersgill, & I. Van Keulen (Eds.), *Sociological reflections on the neurosciences (Vol. Advances in medical Sociology 13* (pp. 203–228). Emerald Group: Bingley.

[CIT0007] De Bruyn, S., Wouters, E., Ponnet, K., & Van Hal, G. (2019). Popping smart pills in medical school: Are competition and stress associated with the misuse of prescription stimulants among students? *Substance Use & Misuse*, *54*(7), 1191–1202. doi:10.1080/10826084.2019.1572190 30892122

[CIT0008] DeSantis, A. D., & Hane, A. C. (2010). “Adderall is definitely not a drug”: justifications for the illegal use of ADHD stimulants. *Substance Use and Misuse*, *45*(1-2), 31–46. doi:10.3109/10826080902858334 20025437

[CIT0009] Dundas, M. L., & Keller, J. R. (2003). Herbal, vitamin, and mineral supplement Use and beliefs of University students. *Topics in Clinical Nutrition*, *18*(1), 49–53. doi:10.1097/00008486-200301000-00007

[CIT0010] Forlini, C., & Racine, E. (2009). Autonomy and coercion in academic “cognitive enhancement” using methylphenidate: Perspectives of key stakeholders. *Neuroethics*, *2*(3), 163–177. doi:10.1007/s12152-009-9043-y

[CIT0011] Forlini, C., & Racine, E. (2012). Added stakeholders, added value (s) to the cognitive enhancement debate: Are academic discourse and professional policies sidestepping values of stakeholders? *AJOB Primary Research*, *3*(1), 33–47. doi:10.1080/21507716.2011.645116

[CIT0012] Forlini, C., Schildmann, J., Roser, P., Beranek, R., & Vollmann, J. (2015). Knowledge, experiences and views of German university students toward neuroenhancement: An empirical-ethical analysis. *Neuroethics*, *8*(2), 83–92. doi:10.1007/s12152-014-9218-z

[CIT0013] Franke, A. G., Lieb, K., & Hildt, E. (2012). What users think about the differences between caffeine and illicit/prescription stimulants for cognitive enhancement. *PLoS ONE*, *7*(6), e40047. doi:10.1371/journal.pone.0040047 22768218 PMC3386931

[CIT0014] Garasic, M. D., & Lavazza, A. (2016). Moral and social reasons to acknowledge the use of cognitive enhancers in competitive-selective contexts. *BMC Medical Ethics*, *17*(1), 18. doi:10.1186/s12910-016-0102-8 27025299 PMC4812634

[CIT0015] Hall, K. M., Irwin, M. M., Bowman, K. A., Frankenberger, W., & Jewett, D. C. (2005). Illicit use of prescribed stimulant medication among college students. *Journal of American College Health*, *53*(4), 167–174. doi:10.3200/Jach.53.4.167-174 15663065

[CIT0016] Hildt, E., Lieb, K., & Franke, A. G. (2014). Life context of pharmacological academic performance enhancement among university students – a qualitative approach. *BMC Medical Ethics*, *15*(1), 23. doi:10.1186/1472-6939-15-23 24606831 PMC3973848

[CIT0017] International Narcotics Control Board. (2013). *Report of the International Narcotics Control Board for* 2012. Retrieved from New York: https://www.incb.org/documents/Publications/AnnualReports/AR2012/AR_2012_E.pdf

[CIT0018] Johnson, S. K., & Blanchard, A. (2006). Alternative medicine and herbal Use Among University students. *Journal of American College Health*, *55*(3), 163–168. doi:10.3200/Jach.55.3.163-168 17175902

[CIT0019] Judson, R., & Langdon, S. W. (2009). Illicit use of prescription stimulants among college students: Prescription status, motives, theory of planned behaviour, knowledge and self-diagnostic tendencies. *Psychology, Health & Medicine*, *14*(1), 97–104. doi:10.1080/13548500802126723 19085316

[CIT0020] Kieffer, K. M., Cronin, C., & Gawet, D. L. (2006). Test and study worry and emotionality in the prediction of college students’ reasons for drinking: An exploratory investigation. *Journal of Alcohol and Drug Education*, *50*(1), 57–81.

[CIT0021] Lee, K. H., Human, G. P., Fourie, J. J., Louw, W. A. N., Larson, C. O., & Joubert, G. (2009). Medical students’ use of caffeine for ‘academic purposes’ and their knowledge of its benefits, side-effects and withdrawal symptoms. *South African Family Practice*, *51*(4), 322–327. doi:10.1080/20786204.2009.10873872

[CIT0022] Leonard, B. E., McCartan, D., White, J., & King, D. J. (2004). Methylphenidate: A review of its neuropharmacological, neuropsychological and adverse clinical effects. *Human Psychopharmacology*, *19*(3), 151–180. doi:10.1002/hup.579 15079851

[CIT0023] Mache, S., Eickenhorst, K., Vitzthum, K., Klapp, B. F., & Groneberg, D. A. (2012). Cognitive-enhancing substance use at German universities: Frequency, reasons and gender differences. *Wiener Medizinische Wochenschrift*, *162*(11-12), 262–271. doi:10.1007/s10354-012-0115-y 22707077

[CIT0024] Maier, L. J., Liakoni, E., Schildmann, J., Schaub, M. P., & Liechti, M. E. (2015). Swiss University students’ attitudes toward pharmacological cognitive enhancement. *PLoS ONE*, *10*(12), e0144402. doi:10.1371/journal.pone.0144402 26657300 PMC4675521

[CIT0025] Mazanov, J., Dunn, M., Connor, J., & Fielding, M.-L. (2013). Substance use to enhance academic performance among Australian university students. *Performance Enhancement & Health*, *2*(3), 110–118. doi:10.1016/j.peh.2013.08.017

[CIT0026] McCabe, S. E., Knight, J. R., Teter, C. J., & Wechsler, H. (2005). Non-medical use of prescription stimulants among US college students: Prevalence and correlates from a national survey. *Addiction*, *100*(1), 96–106. doi:10.1111/j.1360-0443.2005.00944.x 15598197

[CIT0027] Moser, L., Dellen, R. G., & Lundt, P. V. (1979). The effect of betadrenol on examination anxiety [Die wirksamkeit von betadrenol bei pruefungsangst]. *Medizinische Klinik*, *74*(41), 1500–1504. 43950

[CIT0028] Newberry, H., Beerman, K., Duncan, S., McGuire, M., & Hillers, V. (2001). Use of nonvitamin, nonmineral dietary supplements among college students. *Journal of American College Health*, *50*(3), 123–129. doi:10.1080/07448480109596016 11765248

[CIT0029] Paliza, J. (2011). Use of cognitive enhancing substances by University students: A cross-sectional study (Unpublished master's thesis), Curtin University of Technology, Australia.

[CIT0030] Parks, K. A., Levonyan-Radloff, K., Przybyla, S. M., Darrow, S., Muraven, M., & Hequembourg, A. (2017). University student perceptions about the motives for and consequences of non-medical use of prescription drugs (NMUPD). *Journal of American College Health*, 00–00. doi:10.1080/07448481.2017.1341895 PMC7874525 28617176

[CIT0031] Partridge, B., Bell, S., Lucke, J., & Hall, W. (2013). Australian university students’ attitudes towards the use of prescription stimulants as cognitive enhancers: Perceived patterns of use, efficacy and safety. *Drug and Alcohol Review*, *32*(3), 295–302. doi:10.1111/dar.12005 23121045

[CIT0032] Perkin, J. E., Wilson, W. J., Schuster, K., Rodriguez, J., & Allen-Chabot, A. (2002). Prevalence of nonvitamin, nonmineral supplement usage among university students. *Journal of the American Dietetic Association*, *102*(3), 412–414. doi:10.1016/S0002-8223(02)90096-9 11902377

[CIT0033] Petersen, M., Nørgaard, L., & Traulsen, J. (2015). Pursuing pleasures of productivity: University Students’ Use of prescription stimulants for enhancement and the moral uncertainty of making work Fun. *Culture, Medicine, and Psychiatry*, 1–15. doi:10.1007/s11013-015-9457-4 25956594

[CIT0034] Petersen, M. A., Lyngsø-Dahl Ølgaard, L. J., & Nørgaard, L. S. (2019). Contextualizing study drugs - An exploratory study of perceptions and practices among study counselors, general practitioners, psychiatrists and from student polls. *Research in Social and Administrative Pharmacy*, *15*(10), 1204–1211. doi:10.1016/j.sapharm.2018.10.005 30448283

[CIT0035] Petersen, M. A., Petersen, I. L., Poulsen, C., & Nørgaard, L. S. (2021). #Studydrugs–persuasive posting on instagram. *International Journal of Drug Policy*, *103100*, doi:10.1016/j.drugpo.2020.103100 33451861

[CIT0036] Ram, S., Hussainy, S., Henning, M., Jensen, M., & Russell, B. (2015). Prevalence of cognitive enhancer use among New Zealand tertiary students. *Drug and Alcohol Review*, *35*(3), 345–351. doi:10.1111/dar.12294 26121209

[CIT0037] Ram, S., Hussainy, S., Henning, M., Stewart, K., Jensen, M., & Russell, B. R. (2017). Attitudes toward cognitive enhancer Use Among New Zealand tertiary students. *Substance Use & Misuse*, *52*(11), 1387–1392. doi:10.1080/10826084.2017.1281313 28429997

[CIT0038] Ram, S., Russell, B. R., Stewart, K., Kirkpatrick, C., Henning, M., Scahill, S., & Hussainy, S. (2021). Psychiatrists’ attitudes towards and willingness to prescribe cognitive enhancers in academic settings. *Drugs: Education, Prevention and Policy*, *28*(1), 59–66. doi:10.1080/09687637.2020.1735303

[CIT0039] Riddell, C., Jensen, C., & Carter, O. (2018). Cognitive enhancement and coping in an Australian University student sample. *Journal of Cognitive Enhancement*, *2*(1), 63–69. doi:10.1007/s41465-017-0046-z

[CIT0040] Schelle, K. J., Olthof, B. M. J., Reintjes, W., Bundt, C., Gusman-Vermeer, J., & van Mil, A. C. C. M. (2015). A survey of substance use for cognitive enhancement by university students in the Netherlands. *Frontiers in Systems Neuroscience*, *9*(10), doi:10.3389/fnsys.2015.00010 PMC4330699 25741248

[CIT0041] Sharif, S., Guirguis, A., Fergus, S., & Schifano, F. (2021). The Use and impact of cognitive enhancers among University students: A systematic review. *Brain Sciences*, *11*(3), 355. 33802176 10.3390/brainsci11030355 PMC8000838

[CIT0042] Simoni-Wastila, L., & Strickler, G. (2004). Risk factors associated With problem Use of prescription drugs. *American Journal of Public Health*, *94*(2), 266–268. doi:10.2105/Ajph.94.2.266 14759941 PMC1448242

[CIT0043] Smith, M. E., & Farah, M. J. (2011). Are prescription stimulants “smart pills”? The epidemiology and cognitive Neuroscience of prescription stimulant Use by normal healthy individuals. *Psychological Bulletin*, *137*(5), 717–741. doi:10.1037/a0023825 21859174 PMC3591814

[CIT0044] Solomon, P. R., Adams, F., Silver, A., Zimmer, J., & DeVeaux, R. (2002). Ginkgo for memory enhancement: A randomized controlled trial. *Journal of the American Medical Association*, *288*(7), 835–840. doi:10.1001/jama.288.7.835 12186600

[CIT0045] Stasio, M. J., Curry, K., Sutton-Skinner, K. M., & Glassman, D. M. (2010). Over-the-Counter medication and herbal or dietary supplement Use in college: Dose frequency and relationship to self-reported distress. *Journal of American College Health*, *56*(5), 535–548. doi:10.3200/JACH.56.5.535-548 18400666

[CIT0046] Teter, C. J., McCabe, S. E., Cranford, J. A., Boyd, C. J., & Guthrie, S. K. (2005). Prevalence and motives for illicit Use of prescription stimulants in an Undergraduate student sample. *Journal of American College Health*, *53*(6), 253–262. 15900989 10.3200/JACH.53.6.253-262

[CIT0047] Urban, K. R., & Gao, W.-J. (2017). Psychostimulants As cognitive enhancers in adolescents: More risk than reward? *Frontiers in Public Health*, *5*(260), doi:10.3389/fpubh.2017.00260 PMC5626934 29034227

[CIT0048] Vuckovic, N. (1999). Fast belief: Buying time with medications. *Medical Anthropology Quarterly*, *13*(1), 51–68. doi:10.1525/maq.1999.13.1.51 10322601

[CIT0049] White, B. P., Becker-Blease, K. A., & Grace-Bishop, K. (2006). Stimulant medication Use, misuse, and abuse in an Undergraduate and graduate student sample. *Journal of American College Health*, *54*(5), 261–268. doi:10.3200/JACH.54.5.261-268 16539218

[CIT0050] Williams, S. J., Martin, P., & Gabe, J. (2011). The pharmaceuticalisation of society? A framework for analysis. *Sociology of Health and Illness*, *33*(5), 710–725. doi:10.1111/j.1467-9566.2011.01320.x 21371048

