# Supplementary material for: Students’ use of caffeine, alcohol, dietary supplements, and illegal substances for improving academic performance in a New Zealand university
Source: Health Psychol Behav Med. 2021 Oct 22;9(1):917–32. doi: 10.1080/21642850.2021.1990763 (PMC8547821; doi:10.1080/21642850.2021.1990763)
Supplement: Supplemental Material [file RHPB_A_1990763_SM2982.docx]

Table B1. Concurrent 6 month use of substances for improving academic performance

|  | Caffeine use  n (%) | Alcohol use  n (%) | Dietary substances use  n (%) | Prescription stimulants use  n (%) | Other prescription substances use  n (%) | Illicit substances use  n (%) |
| --- | --- | --- | --- | --- | --- | --- |
| Caffeine use | 416 | 20 (80) | 106 (66) | 11 (100) | 11 (73) | 16 (84) |
| Alcohol use | 20 (5) | 25 | 8 (5) | 2 (18) | 3 (20) | 1 (5) |
| Dietary substance use | 106 (25) | 8 (32) | 161 | 3 (27) | 7 (47) | 7 (37) |
| Prescription stimulants use | 11 (3) | 2 (8) | 3 (2) | 11 | 6 (40) | 0 (0) |
| Other prescription substances use | 11 (3) | 3 (12) | 7 (4) | 6 (55) | 15 | 3 (16) |
| Illicit substances use | 16 (4) | 1 (4) | 7 (4) | 0 (0) | 3 (20) | 19 |

Note: Includes only those who reported the use of at least one substance in the last 6 months. Percentages reflect the proportion of current 6 month users of the column heading that also used the substance in the row heading in the preceding 6 months.

Table B.2. Univariate odds ratios showing relationship between demographic factors and use of substances in last 6 months.

|  | Caffeine | Alcohol | Dietary Supplements | All controlled substances |
| --- | --- | --- | --- | --- |
| Gender |  |  |  |  |
| Male | [ref] |  |  |  |
| Female | .87(0.62-1.20) | **0.37(0.17-0.84)** | **1.75(1.17-2.62)** | **0.31(0.17-0.59)** |
| Age (yrs) |  |  |  |  |
| 18-20 | (ref.) |  |  |  |
| 21-23 | 1.01(0.72-1.41) | 1.13(0.44-2.90) | 0.94(0.64-1.39) | 1.53(0.76-3.10) |
| 24-26 | 1.42(0.76-2.67) | 2.79(0.84-9.26) | 0.98(0.49-1.95) | 1.71(0.55-5.34) |
| 27+ | 0.75(0.42-1.32) | 1.86(0.49-6.97) | 0.96(0.49-1.87) | 1.97(0.70-5.62) |
| Year of study |  |  |  |  |
| First | (ref.) |  |  |  |
| Second | 1.42(0.90-2.24) | 1.47(0.41-5.31) | 1.10(0.66-1.84) | 1.79(0.58-5.46) |
| Third | **1.67(1.07-2.61)** | 1.09(0.29-4.15) | 0.99(0.59-1.64) | 1.79(0.60-5.36) |
| Fourth | 1.34(0.80-2.25) | 1.58(0.39-6.47) | 0.85(0.46-1.57) | 2.63(0.83-8.27) |
| Fifth | 1.48(0.79-2.77) | 1.34(0.24-7.50) | 0.58(0.26-1.29) | 2.20(0.57-8.52) |
| 6th | 0.58(0.20-1.70) | 5.65(0.94-33.85) | 0.22(0.03-1.73) | 2.09(0.23-19.13) |
| 7+ | 1.53(0.72-3.24) | 2.16(0.38-12.29) | 1.19(0.52-2.69) | **4.71(1.29-17.26)** |
| Division^1^ |  |  |  |  |
| Humanities | 1.10(0.79-1.54) | 1.31(0.57-3.02) | 1.22(0.84-1.78) | **2.58(1.35-4.95)** |
| Business | 1.10(0.68-1.76) | no users | 1.12(0.66-1.90) | 1.07(0.41-2.82) |
| Sciences | 0.81(0.59-1.12) | 1.21(0.53-2.73) | 1.02(0.70-1.47) | 0.60(0.29-1.26) |
| Health Sci. | 1.09(0.78-1.52) | 0.73(0.29-1.85) | 0.78(0.52-1.15) | 0.68(0.32-1.47) |
| Competitive courses^1^ | |  |  |  |
| Medicine | 1.20(0.74-1.96) | 0.66(0.15-2.85) | 0.61(0.33-1.14) | 1.04(0.40-2.73) |
| Law | 1.40(0.83-2.35) | 0.35(0.05-2.64) | 1.12(0.63-1.97) | 1.18(0.45-3.11) |
| HSFY | 0.88(0.48-1.62) | 0.58(0.08-4.41) | 0.80(0.38-1.70) | no users |
| Notes ^1^ For these analyses, each subject course group was compared to all other students in separate analyses, due to potentially overlapping categories.  Bolded values indicate CI does not include 1.0 | | | | |

Table B.3. Times of use by substance for improving academic performance (current users only)

| Time^1^ | Caffeine | Alcohol | Dietary supplements | Prescription Stimulants | Other prescription substances | Illicit substances for academic purpose |
| --- | --- | --- | --- | --- | --- | --- |
| Before assignments deadlines | 306 | 12 | 42 | 6 | 2 | 11 |
| Before mid-semester tests | 218 | 0 | 44 | 2 | 0 | 5 |
| Before final exams | 290 | 1 | 70 | 6 | 1 | 6 |
| Other times | 57 | 10 | 19 | 1 | 2 | 8 |
| **All current users (i.e., any of above)** | **416** | **25**^2^ | **161** | **11** | **5** | **19** |

Notes: ^1^ Multiple times possible, therefore column total can exceeds the "all current users".

^2^ Column total smaller than "all current users" due to some current drinkers not specifying "time".

Table B.4. Reasons given for substance use by substance.

| Reason | Total  % (n=476) | Caffeine  % (n=356) | Alcohol  %  (n=19) | Dietary supplements  %  (n=137) | Prescription stimulants (without ADHD)  %  (n=8) | Other prescription substances  %  (n=13) | Illicit substances  %  (n=16) |
| --- | --- | --- | --- | --- | --- | --- | --- |
| Stay awake | 70 | 89 | 0 | 20 | 50 | 8 | 19 |
| Improve concentration | 57 | 61 | 5 | 57 | 100 | 46 | 31 |
| Improve memory | 15 | 6 | 0 | 39 | 38 | 23 | 13 |
| Relaxation | 26 | 16 | 79 | 35 | 0 | 77 | 100 |
| Because others do it | 4 | 5 | 0 | 4 | 0 | 0 | 0 |
| Inspiration or creativity | 11 | 5 | 42 | 10 | 13 | 16 | 75 |
| To manage the pressure to succeed | 15 | 12 | 32 | 15 | 38 | 39 | 44 |
| Other reason | 19 | 13 | 16 | 36 | 0 | 23 | 13 |
