# Supplementary figures and images for: Students’ use of caffeine, alcohol, dietary supplements, and illegal substances for improving academic performance in a New Zealand university
Source: Health Psychol Behav Med. 2021 Oct 22;9(1):917–32. doi: 10.1080/21642850.2021.1990763 (PMC8547821; doi:10.1080/21642850.2021.1990763)

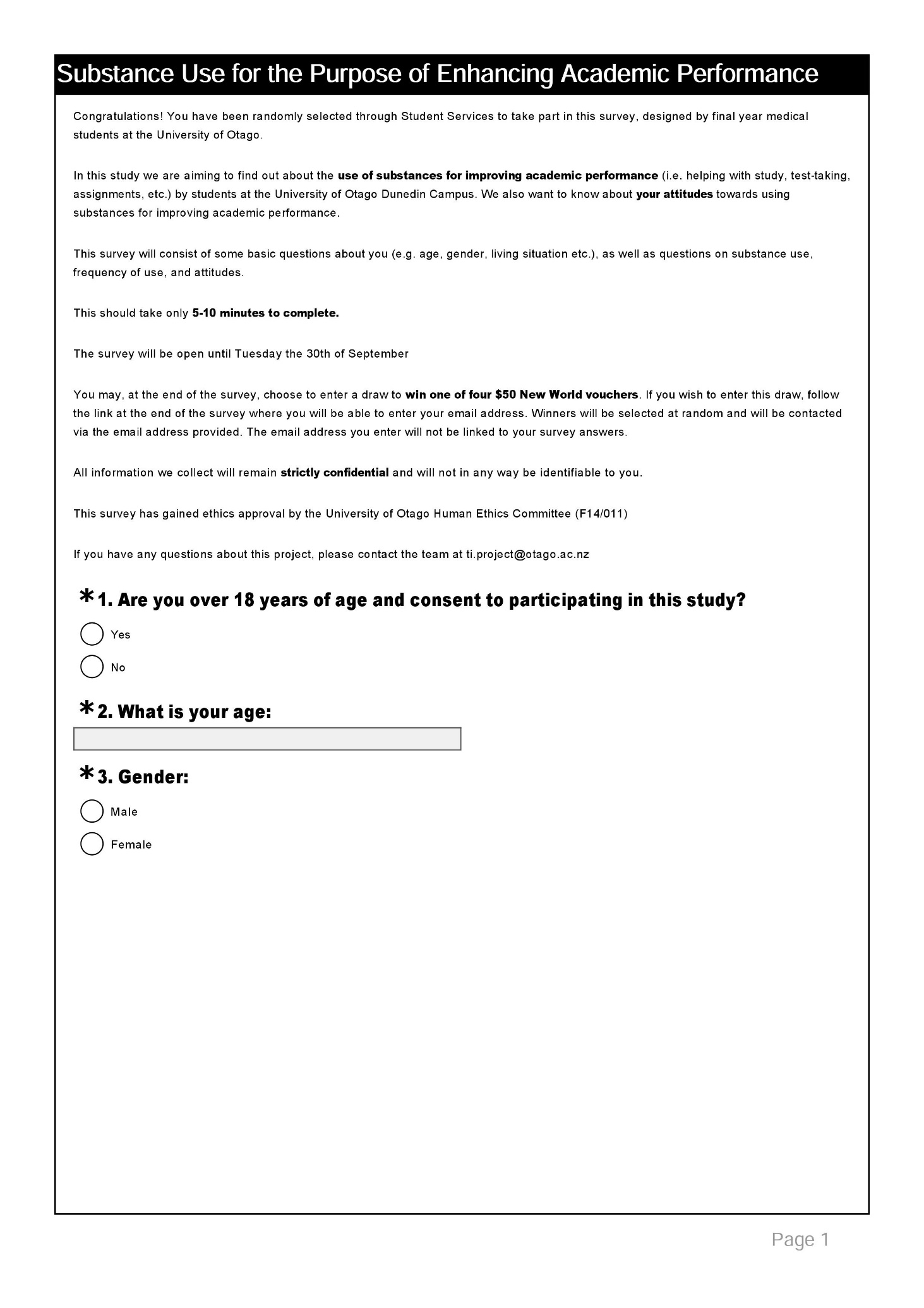

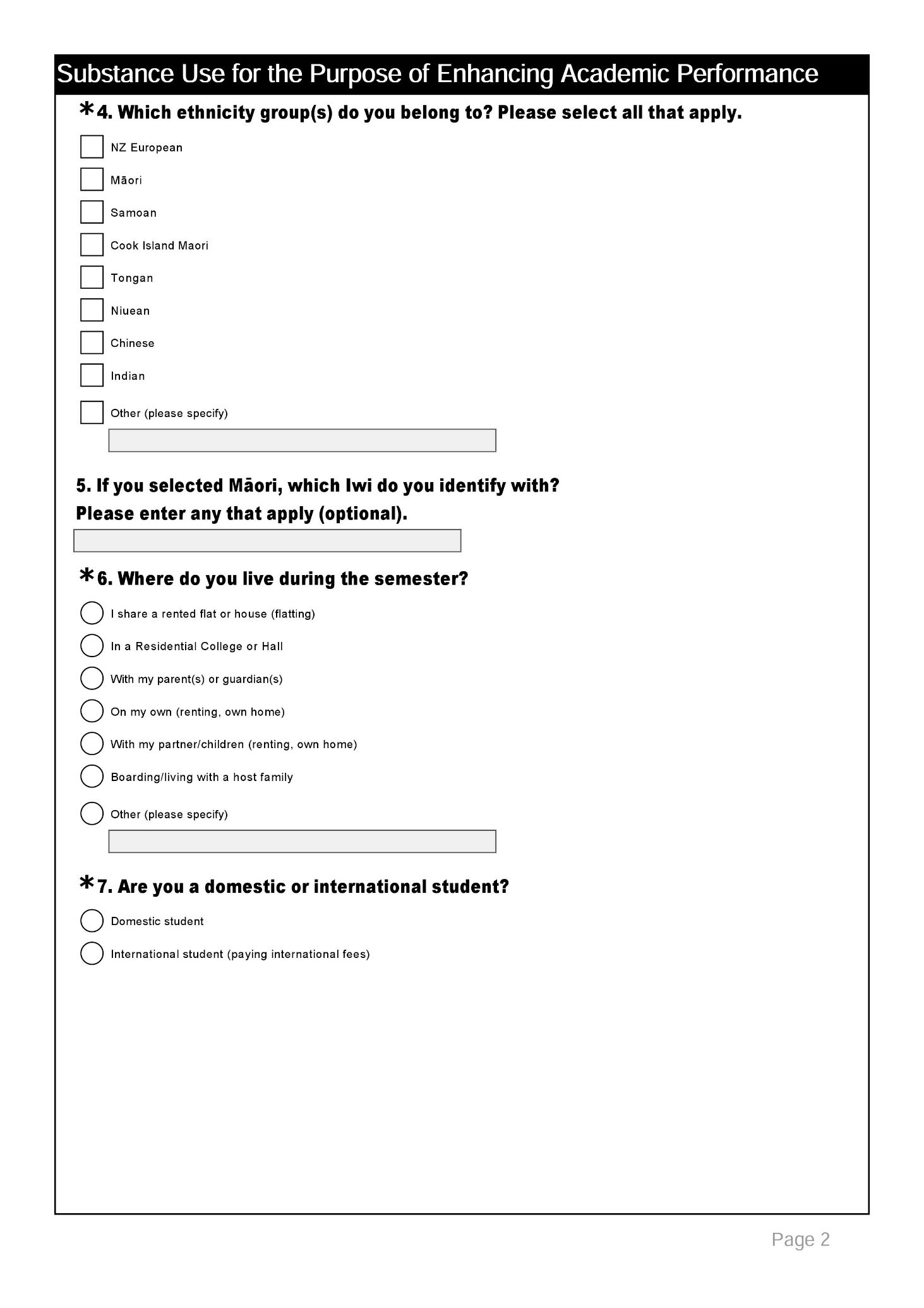

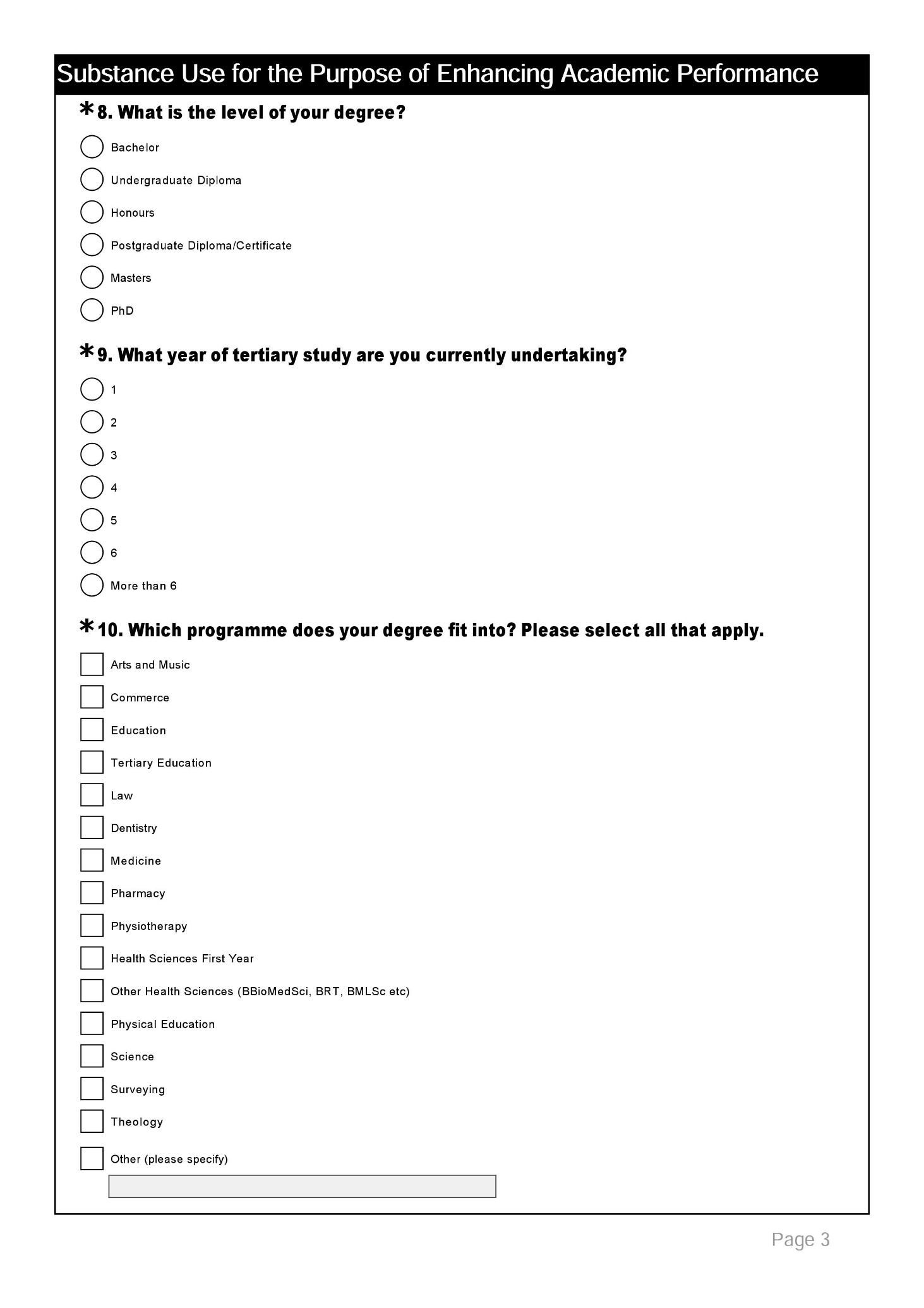

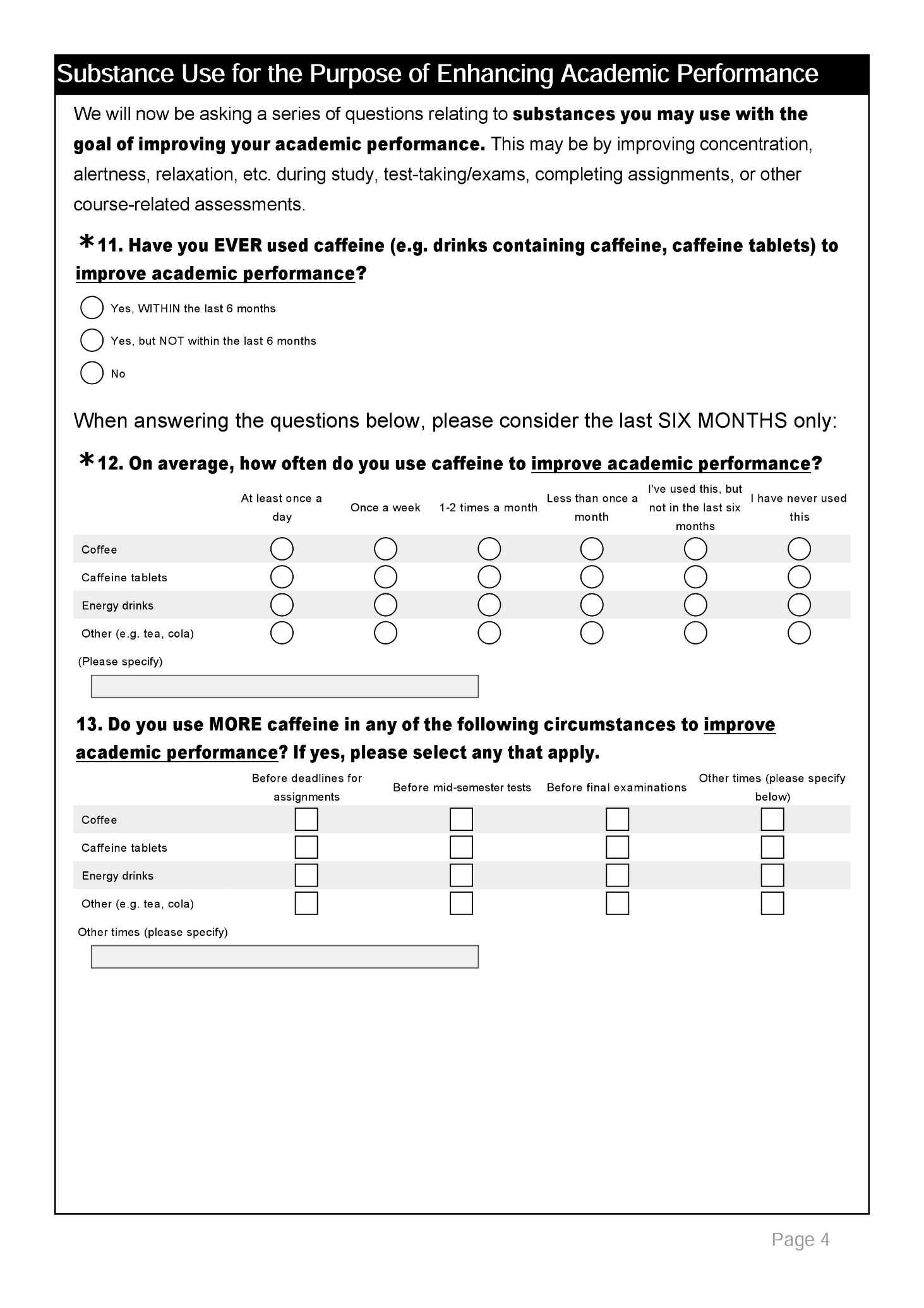

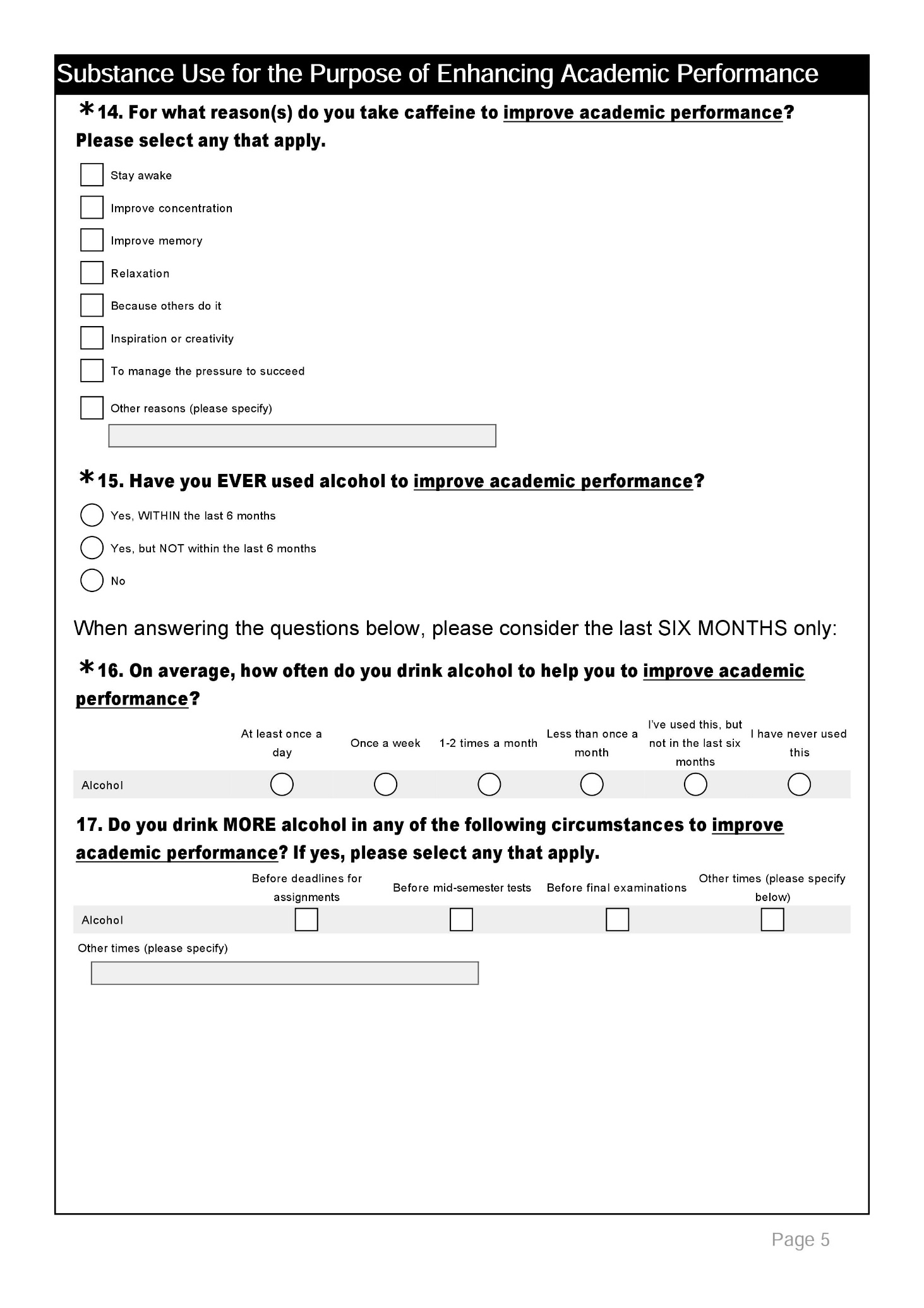

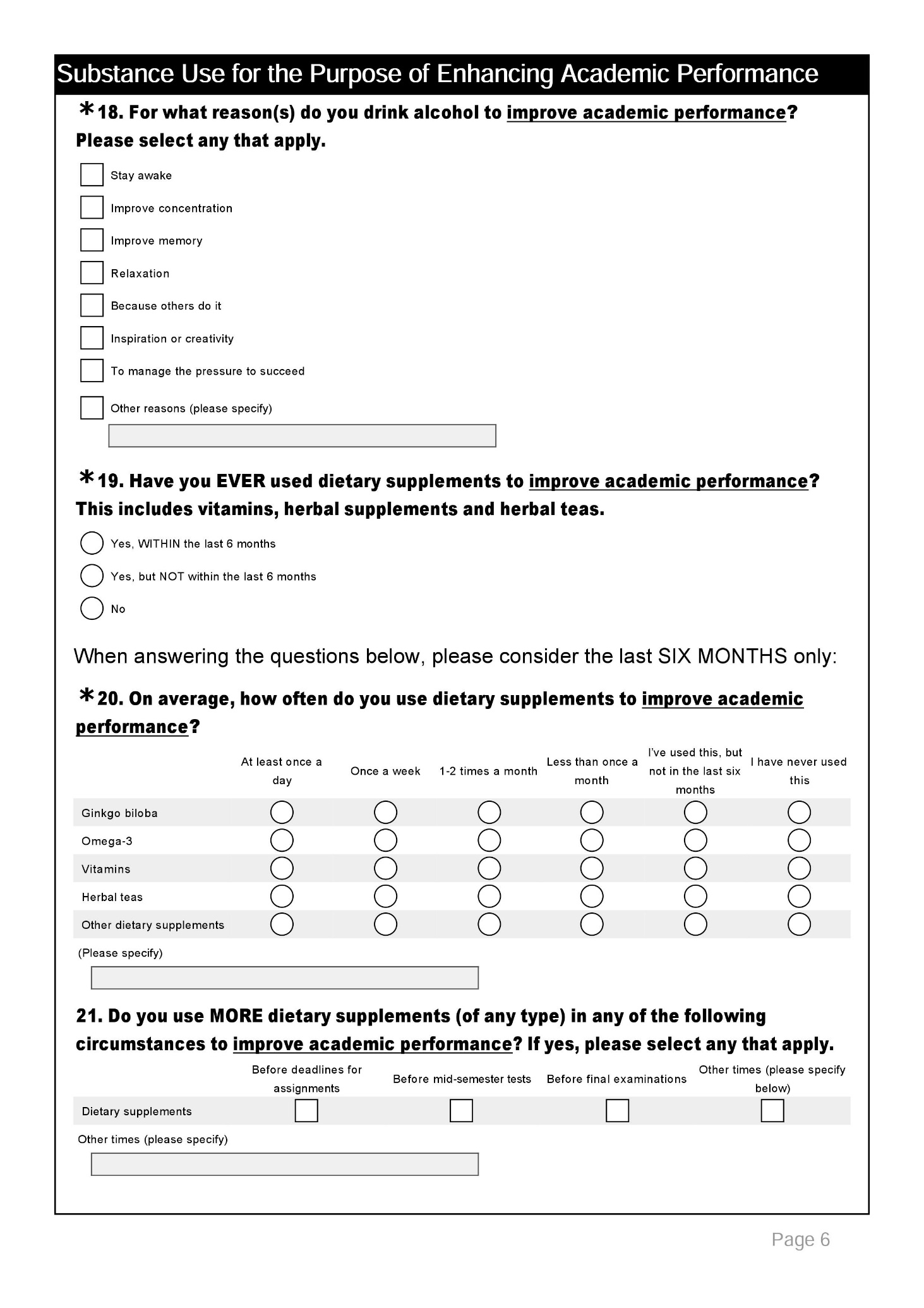

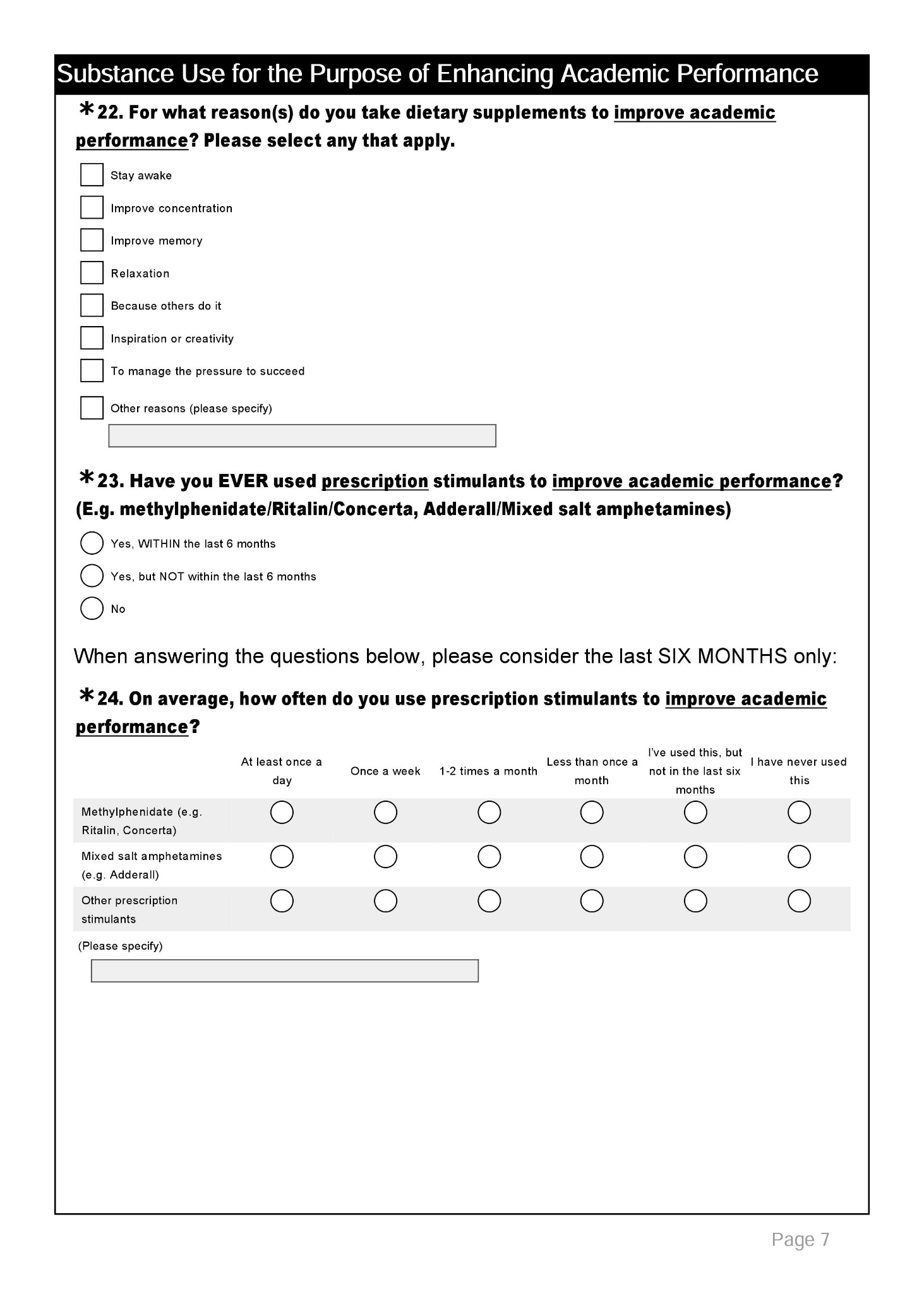

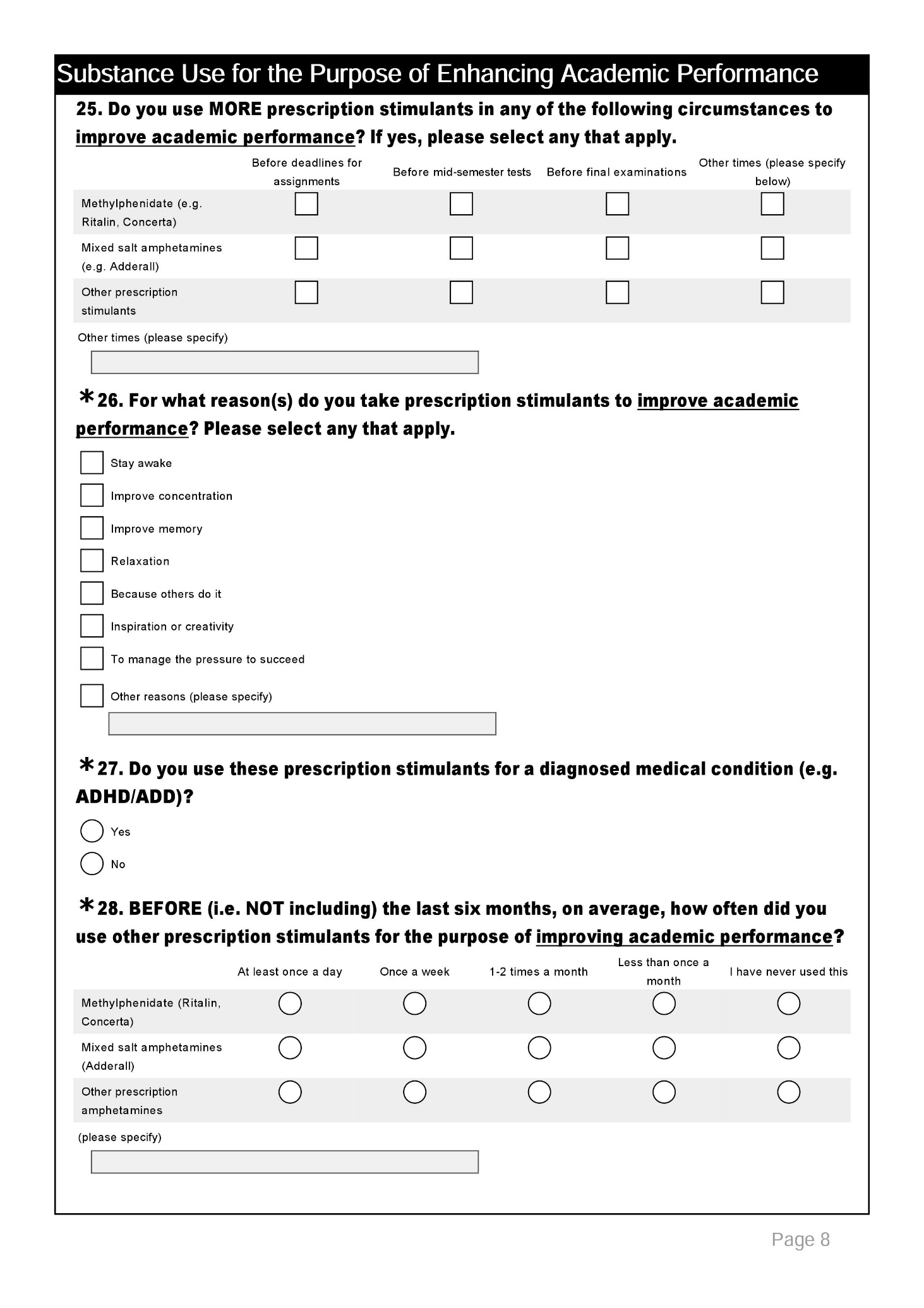

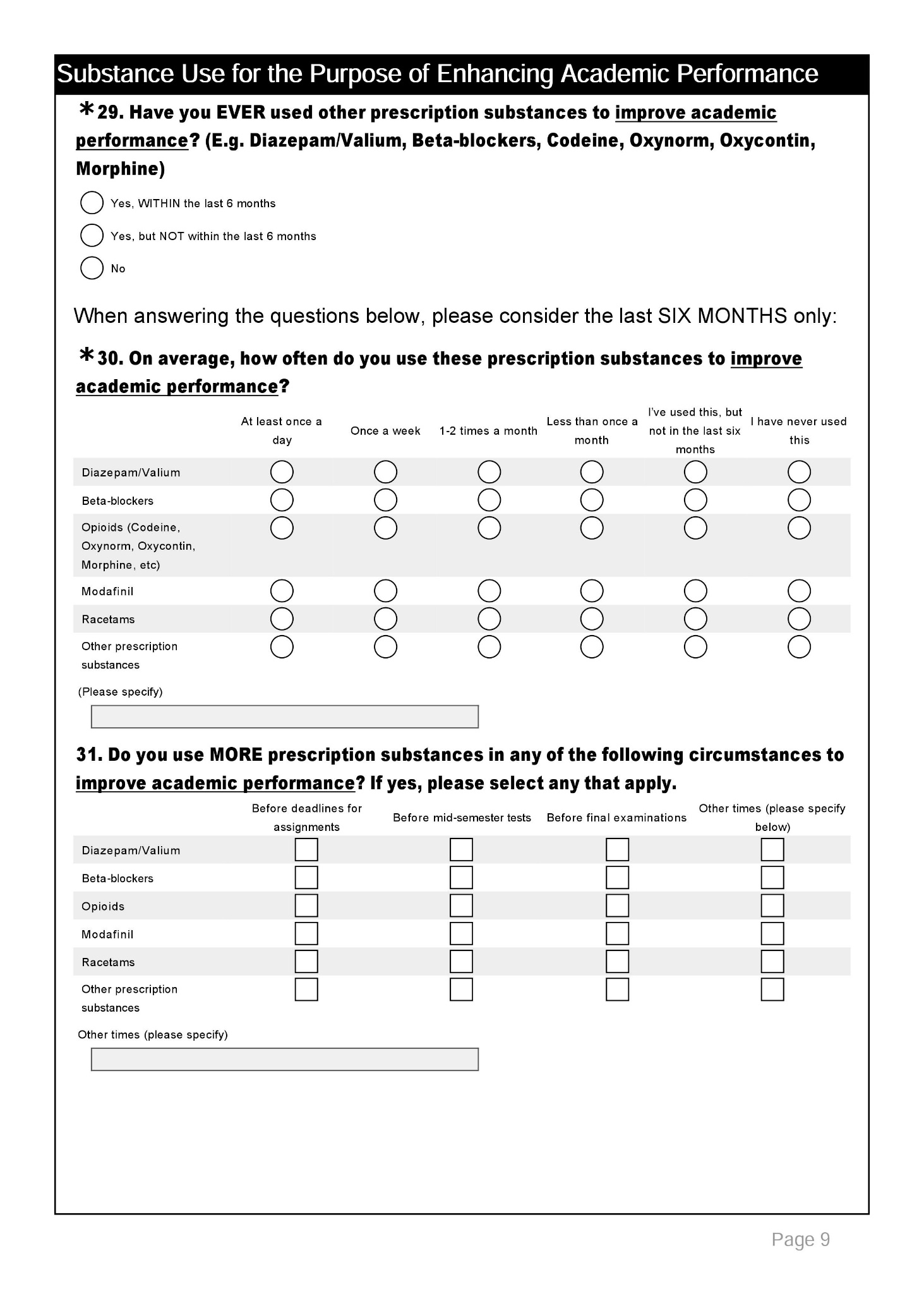

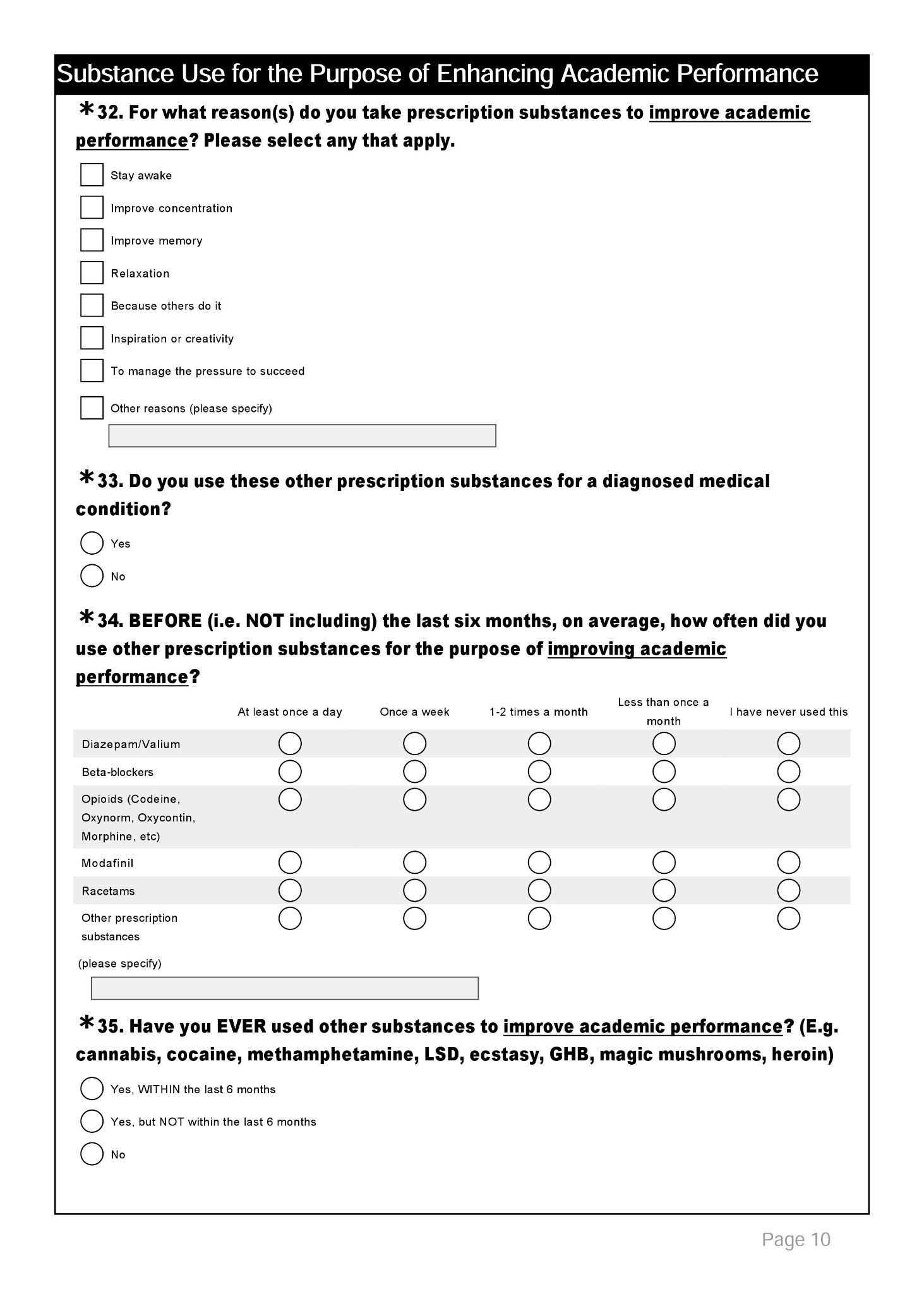

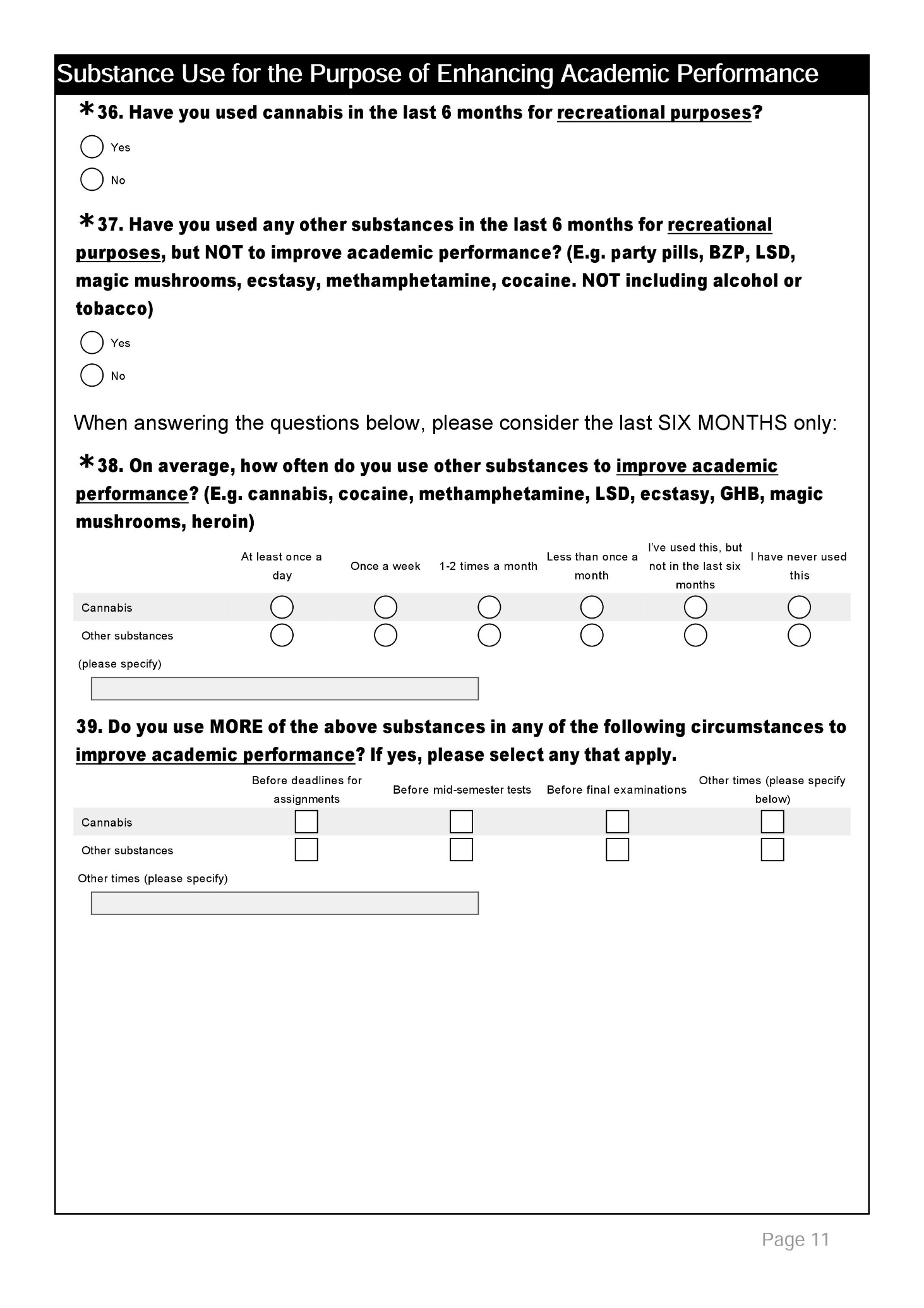

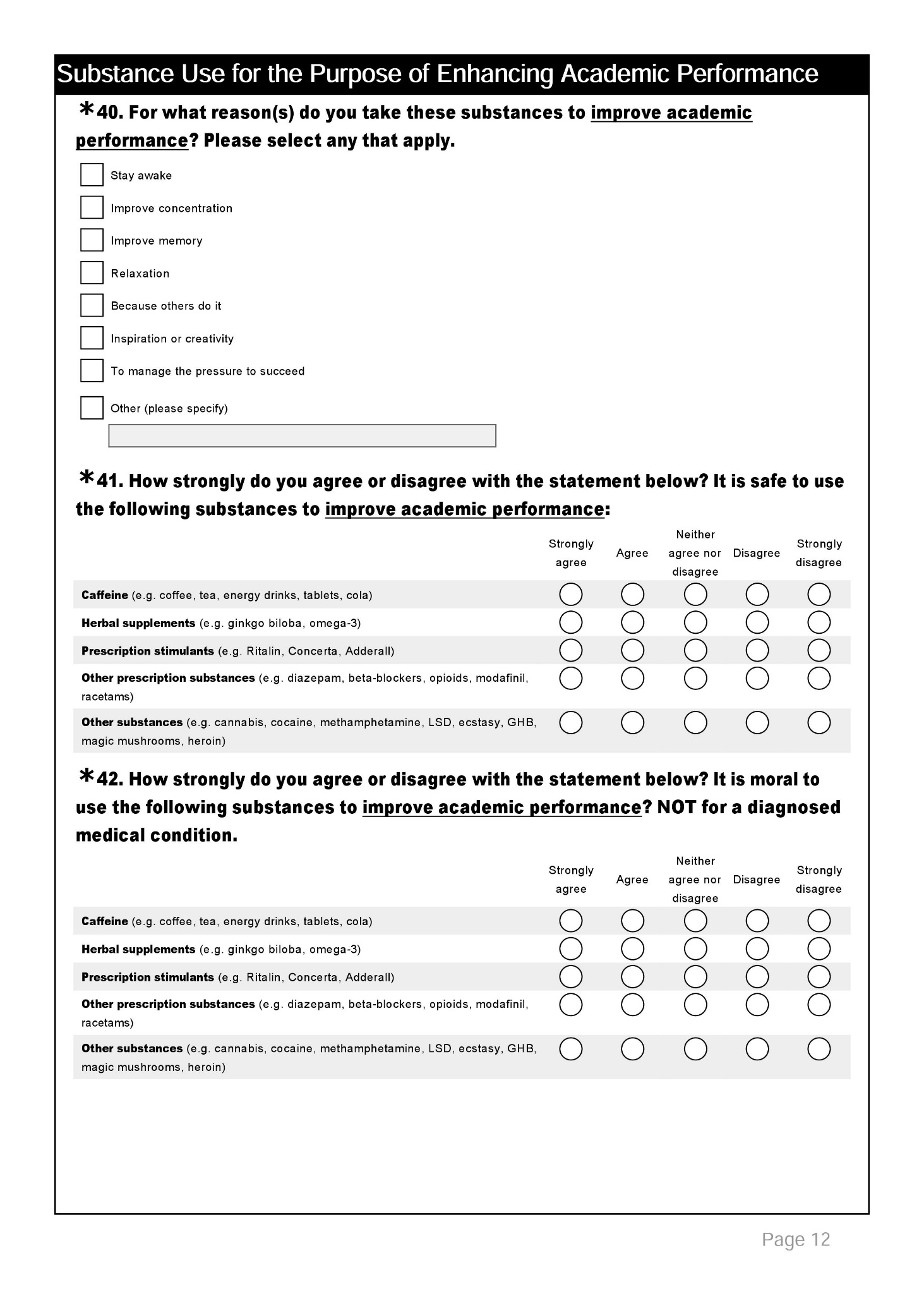

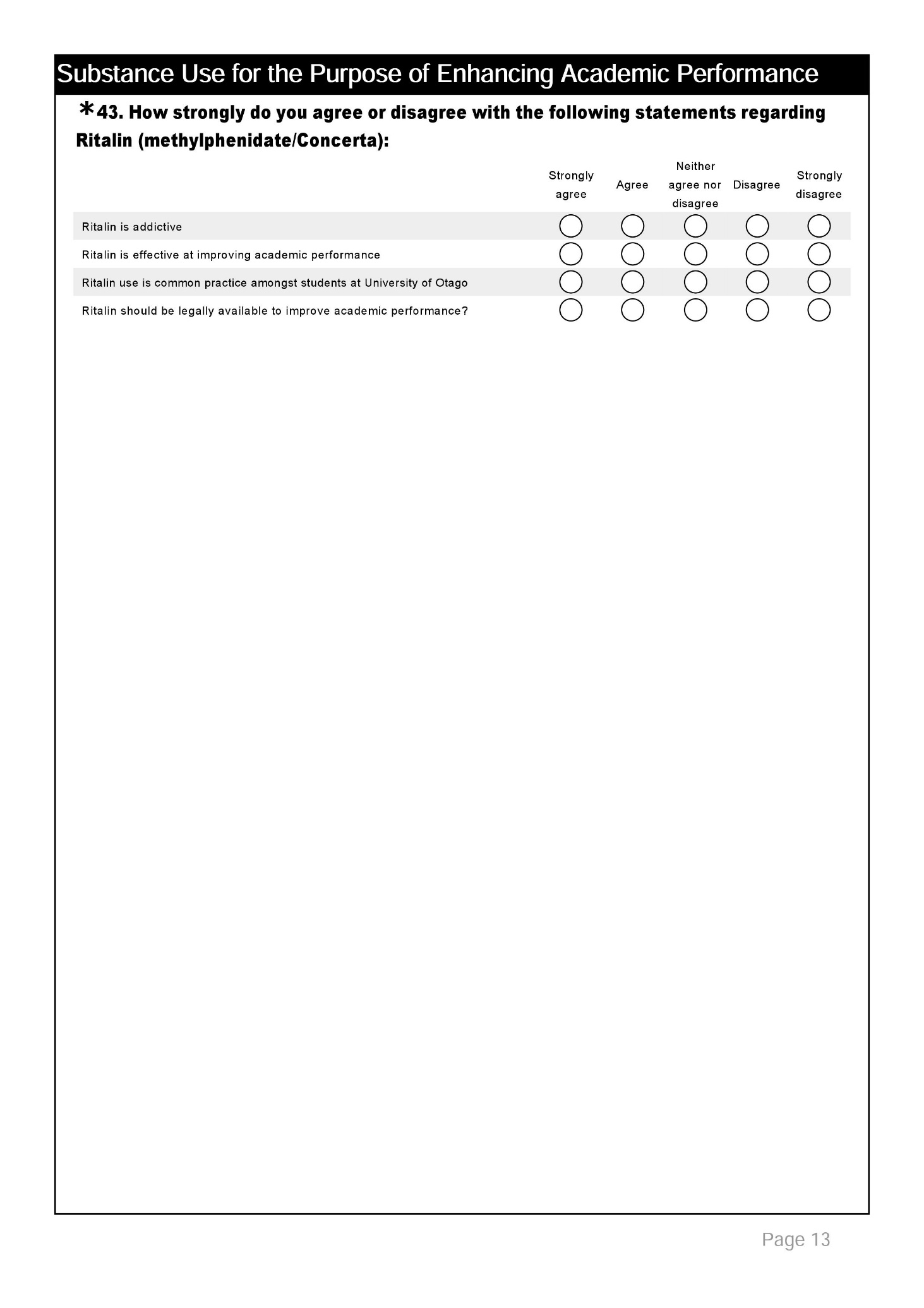

Supplement: Supplemental Material [file RHPB_A_1990763_SM2863.docx]
